# Copy number amplification of TTPAL promotes cholesterol biosynthesis and esophageal squamous cell carcinoma progression via elevating NSUN2-mediated m5C modification of SREBP2 mRNA

**DOI:** 10.1186/s13046-025-03483-8

**Published:** 2025-07-26

**Authors:** Shan Huang, Yuanyuan Liu, Manyu Zhao, Tao Wang, Lihua Mao, Ting Wang, Chunyuan Guo, Wentao Huang, Zimei Peng, Zhen Zhang, Rui Jiang, Xinrui Ma, Nimei Shen, Jun Rao, Xing Wang, Zhi Zheng, Lixiao Chen

**Affiliations:** 1 Jiangxi Provincial People’s Hospital, The First Affiliated Hospital of Nanchang Medical College, Nanchang, 330006 China; 2 Department of Otolaryngology: Head and Neck Surgery, Shanghai General Hospital, Shanghai Jiao Tong University School of Medicine, Shanghai, 200080 China; 3 National Engineering Research Center for Nanomedicine, Key Laboratory of Molecular Biophysics of Ministry of Education, College of Life Science and Technology, Department of Oncology, Tongji Hospital, Huazhong University of Science and Technology, Wuhan, 430074 China; 4 Institute of Geriatrics, Jiangxi Provincial People’s Hospital, The First Affiliated Hospital of Nanchang Medical College, Nanchang, 330006 Jiangxi PR China; 5 Institute of Clinical Medicine, Jiangxi Provincial People’s Hospital, The First Affiliated Hospital of Nanchang Medical College, Nanchang, 330006 China; 6 Department of Otolaryngology: Head and Neck Surgery, Nanjing University of Chinese Medicine Affiliated Kunshan Traditional Chinese Medicine Hospital, Jiangsu, 215300 China; 7 Department of Otolaryngology: Head and Neck Surgery, The First People’s Hospital of Kunshan, Jiangsu, 215300 China; 8 Department of Otolaryngology: Head and Neck Surgery, Jiangsu General Hospital, The Second Affiliated Hospital of Nantong University, Jiangsu, 226000 China; 9 The Second Affiliated Hospital of Nanchang Medical College, Jiangxi Cancer Hospital, Jiangxi Clinical Research Center for Cancer, Nanchang, 330006 China; 10 Centre for Medical Research and Translation, Jiangxi Provincial People’s Hospital, The First Affiliated Hospital of Nanchang Medical College, Nanchang, 330006 China

**Keywords:** TTPAL, Esophageal squamous cell carcinoma, SREBP2, Reprogrammed cholesterol metabolism, NSUN2, Simvastatin therapy resistance

## Abstract

Alterations in copy number are crucial genetic events in the development of esophageal squamous cell carcinoma (ESCC). Here, we show that Tocopherol alpha transfer protein-like (*TTPAL*) is highly amplified and frequently overexpressed in human ESCC. Using *Ttpal-*KO mouse mode, we demonstrate that TTPAL promotes ESCC cell proliferation and accelerates tumor development by driving cholesterol biosynthesis. Mechanistically, TTPAL upregulates a key transcription factor in cholesterol biosynthesis-sterol regulatory element-binding transcription factor (SREBP2) in ESCC cells. TTPAL interacts with the RNA methyltransferase NSUN2 and relieves the ubiquitination of NSUN2, protecting NSUN2 from proteasome-mediated degradation. In turn, NSUN2 catalyzes the m5C modification of *SREBP2* mRNA, and then the m5C modified *SREBP2* mRNA binds to the m5C reader protein-ALYREF to enhance its stability, thereby increasing SREBP2 expression. Moreover, we validate the efficacy of cholesterol biosynthesis inhibitor simvastatin in ESCC with high TTPAL expression. Overall, our results uncover a novel function of TTPAL in regulating SREBP2 expression, revealed a previously unknown TTPAL/NSUN2/SREBP2 pathway that promotes cholesterol biosynthesis in ESCC cells, and identified sensitively to cholesterol biosynthesis inhibitor simvastatin.

**Supplementary Information:**

The online version contains supplementary material available at 10.1186/s13046-025-03483-8.

## Introduction

Esophageal squamous cell carcinoma (ESCC) remains one of the most prevalent malignant tumors, with a 5-year survival rate of only approximately 19% [1]. The escalating global incidence and mortality rates have established ESCC as a significant public health challenge, with most patients ultimately succumbing to disease progression owing to limited effective treatment options [2]. Therefore, further exploration of the mechanisms regulating ESCC is important for developing more effective treatment modalities.

Copy number variations (CNVs) are somatic changes in chromosomal structure characterized by submicroscopic DNA alterations between 1 Kbp and 1 Mbp in length, typically by either loss or gain of DNA segments. The altered expression of oncogenes and tumor suppressors caused by CNVs is associated with the progression of various types of cancer [3]. Beyond the well-characterized oncogenes and tumor suppressors (*EGFR*, *CDKN2A/2B*, *CCND1*), emerging studies have identified additional genetic regulators (*CBX4*, *ZNF750*, *CDCA7*, *FAM84B*) that critically contribute to ESCC pathogenesis [4–6]. Genome-wide analyses further detect recurrent focal CNVs containing prognostically significant loci, where specific genomic alterations drive [7]. Systematic integration of somatic mutation profiles and CNVs patterns through pathway enrichment analyses reveal that ESCC progression is orchestrated by dysregulation of six core signaling networks: (1) RTK-Ras-PI3K cascades, (2) JAK-STAT signaling, (3) Akt survival pathways, (4) NOTCH/Wnt developmental circuits, (5) cell cycle checkpoints, and (6) p53 tumor suppressor axis [8–13]. These collective discoveries uncover promising therapeutic targets for developing precision interventions against ESCC. According to previous genome-wide copy number studies, the most recurrent CNVs in ESCC cohorts from Asian and Western countries involve gains on chromosomes 20q11-q13.33 and so on [14]. *MYBL2* gene is located in 20q13.12 and upregulated in ESCC with copy number gains, which promotes ESCC cells proliferation [15]. Tocopherol alpha transfer protein-like (*TTPAL*) gene is also located at 20q13.12 and promotes gastric and colorectal tumorigenesis by activating PI3K/AKT and Wnt/β-catenin signaling [16, 17]. However, the exact function of *TTPAL* in ESCC and its underlying mechanisms remain unexplored.

Aberrations in epigenetic regulation, such as methylation of RNA or acetylation of cytidine, are common hallmarks of tumor development and recurrence. 5-Methylcytosine (m5C) is a well-known and conserved RNA modification that occurs in various RNAs in eukaryotic cells, including mRNA, rRNA, tRNA, and lncRNAs [18]. Currently, there are two known m5C methyltransferases, i.e., NOP2/Sun RNA methyltransferase family member 1–7 (NSUN1-7) and DNA methyltransferase 2 [19]. The two m5C readers include Y-box binding protein (YBX1) and Aly/REF export factor (ALYREF), which mainly enhance RNA stability and regulate RNA nuclear export [20, 21]. NSUN2 is a major methyltransferase that catalyzes the m5C modification of mammalian mRNAs. NSUN2 is dysregulated in various types of cancers through mutations or translational modifications such as lactylation [22]. In patients with ESCC, NSUN2 expression and the levels of m5C modification are elevated promoting ESCC progression. However, there is a limited understanding of how CNVs increase mRNA m5C modification in patients with ESCC at the genetic level.

Most malignancies exhibit dysregulated lipid metabolism, a metabolic reprogramming event that fuels tumor proliferation [23]. Cholesterol-an essential structural and signaling lipid-maintains cellular membrane integrity and is predominantly synthesized via hepatic sterol biosynthesis before systemic lipoprotein transport [24]. Cholesterol is an essential lipid for cell proliferation and migration while also serving as a signaling molecule in cancer. Cellular cholesterol balance is dynamically regulated through four interdependent processes: de novo synthesis (HMGCR/SREBP2 axis), LDL receptor-mediated uptake, ABC transporter-dependent efflux, and enzymatic conversion to oxysterols/bile acids [25–28]. In tumor cells, reprogramming of cholesterol metabolism occurs to elevate cholesterol levels, which could be a precursor of bile acids, vitamin D, steroid hormones, and cell membranes [29]. It plays a crucial role in cancer progression. Targeting cholesterol biosynthesis is a potential strategy for treating numerous cancers and has been widely tested clinically. In patients with ESCC, cholesterol and its metabolites support the growth, metastasis, stemness, and recurrence of cancer cells. Despite accumulating knowledge about the regulation of cholesterol biosynthesis in cancer cells, the precise role of mRNA m5C modification in cholesterol biosynthesis remains largely unexplored.

In the current study, we determined that *TTPAL* was amplified at 20q13.12 locus, and its amplification was positively associated with its expression in ESCC. Mechanistically, TTPAL drives ESCC progression by orchestrating metabolic reprogramming through m5C-mediated stabilization of *SREBP2* mRNA, which amplifies cholesterol biosynthesis and elevates intracellular cholesterol levels. Furthermore, we established a direct protein-protein interaction between TTPAL and NSUN2 in ESCC cells, competitively disrupting the NSUN2-STUB1 complex to suppress ubiquitin-proteasomal degradation of NSUN2. Moreover, inhibition of cholesterol biosynthesis led to tumor suppression in ESCC cells with high TTPAL expression, offering a potential strategy for ESCC treatment. Collectively, these results delineate a novel epigenetic-metabolic axis wherein TTPAL amplification coordinates RNA methylation (via NSUN2 stabilization) and cholesterol overproduction to fuel ESCC progression, establishing a mechanistic link between genomic alterations, epigenetic remodeling, and metabolic vulnerability in esophageal carcinogenesis.

## Materials and methods

### Sample collection

Between March 2017 to December 2019, we collected 88 tumor and adjacent normal tissue samples from patients with ESCC prior to chemotherapy. All samples were obtained from the Shanghai General Hospital. Written informed consent was obtained from all patients, and the study protocol was approved by the institutional ethics committee.

### Cells and transfection

Non-tumorous cell line (Het-1 A) and six ESCC cell lines (TE-1, TE-10, KYSE30, KYSE180, KYSE150, KYSE510) cell lines were offered by Typical Culture Cell Bank, Chinese Academy of Sciences (Shanghai, China). These cell lines were cultured in RPMI 1640 supplemented with 10% fetal bovine serum (FBS), 100 U/mL penicillin, and 100 µg/mL streptomycin. Bone marrow cells were isolated from mouse femurs and cultured in DMEM supplemented with 20% fetal bovine serum, 1% streptomycin/penicillin, and 10 µM β-mercaptoethanol. For differentiation, cells were treated with either M-CSF (10 ng/ml, Peprotech) to generate bone marrow-derived macrophages (BMDMs) or GM-CSF (20 ng/ml, Peprotech) to generate bone marrow-derived dendritic cells (BMDCs). Cells were maintained at 37 °C in a humidified atmosphere containing 5% CO_2_. For transient transfection, cells were grown to 70–80% confluence, then transfected with plasmids encoding the target gene or the corresponding empty vector controls using Lipofectamine 3000 reagent (L3000001; Thermo Fisher Scientific) following the manufacturer’s instructions.

### Immunohistochemistry (IHC)

Formalin-fixed paraffin-embedded (FFPE) tissue sections from ESCC tumor samples were deparaffinized in xylene for 10 min and rehydrated using a series of ethanol washes (95%, 85%, and 75% ethanol for 5 min for twice), followed by the heat-mediated antigen retrieval using microwave. Tissue sections were blocked with 5% goat blocking serum at room temperature for 30 min to reduce nonspecific antibody binding, then incubated with primary antibodies specific to the target proteins at 4 °C overnight. Sections were incubated with a labeled secondary antibody conjugated to horseradish peroxidase at room temperature for 30 min. Finally, the slides were incubated in ABC reagent for 30 min (the ABC kit, Pierce) and stained with DAB and counterstained with hematoxylin. Stained tissue sections were examined under a light microscope. The staining intensity score was categorized into four grades: negative (0), weak (1), moderate (2) and strong (3). The positive area assessment was stratified using the following criteria: 0 (≤ 10%), 1 (11–25%), 2 (26–50%), 3 (51–75%) and 4 (> 75%). Three experienced pathologists independently evaluated both staining intensity and positive area scores in a blinded manner to clinical data. Immunohistochemical (IHC) scores were subsequently calculated by multiplying the quantitative staining intensity score with the corresponding positive area score for comparative analysis of target protein expression across tumor specimens. For esophageal tissues from mice, H&E was also conducted.

### Fluorescence in situ hybridization and assessment

The bacterial artificial chromosome (BAC) clone RP1-179M20 containing *TTPAL* gene was labeled using Spectrum-Red fluorescent dyes. A chromosome 20 centromere-specific (chromosome enumeration probe, CEP20) probe labeled with Spectrum-Green was utilized as an internal control following previously established protocols. Dual-color FISH assay was performed on 5 μm-thick sections probes to evaluate *TTPAL* amplification following established laboratory protocols described previously. FISH copy number analysis was independently conducted by two pathologists blinded to patients’ clinicopathologic characteristics using a fluorescence microscope. At least 30 tumor cell nuclei per ESCC sample were assessed through signal enumeration (red for *TTPAL* and green for *CEP20*) under oil immersion objective at 1,000× magnification. Overlapping tumor nuclei were excluded to prevent false-positive scoring, ensuring accurate signal quantification. For each case, the average copy numbers of *TTPAL* and *CEP20* signals per tumor cell nucleus were calculated alongside the TTPAL/CEP20 ratio. *TTPAL* amplification was defined as a TTPAL/CEP20 ratio ≥ 2.0; an average *TTPAL* copy number per tumor cell nucleus ≥ 5.0; or ≥ 10% of tumor cells exhibiting large *TTPAL* signal clusters.

### RNA extraction, qRT-PCR analysis, and transcriptome sequencing analysis

Total RNA was harvested from ESCC cells and tissues obtained from patient with ESCC using RNAiso Plus (Cat# 9108, Takara) as described previously. PrimeScript Reverse Transcription Master Mix (Cat# RR036A, Takara) was used to synthesize cDNA by reverse transcription of RNA according to the manufacturer’s instructions. RT-PCR was performed on an Applied Biosystems 7500 system (Foster City, CA, USA) using a TB Green PCR Premium Ex Taq II kit (Cat# RR820A, Takara). The *β-actin* was used as an internal reference and the 2^–ΔΔCt^ method was applied to measure gene relative expression. Primers used in this study are listed in Supplementary Table S1. For transcriptome sequencing analysis, total RNA was extracted from shNC-and shTTPAL-KYSE180 cells according to the method described in our previous study [30].

### Western blotting

Cells were lysed in RIPA buffer (50 mM Tris-HCl, pH 8.0, 150 mM sodium chloride,1% NP-40,0.5% sodium deoxycholate, 0.1%sodium dodecyl sulfate, and 2 mM EDTA) supplemented with protease (Cat# 11836153001, Sigma-Aldrich) and phosphatase inhibitors (Cat# 4906837001, Sigma-Aldrich). Western blotting (WB) was performed according to the standard protocol [31]. Briefly, protein quantification was performed using the Bradford assay (Cat# P0006, Beyotime) following the manufacturer’s protocol. Protein lysates were separated via SDS-PAGE and electrophoretically transferred onto polyvinylidene fluoride (PVDF) membranes. Membranes were blocked with 5% BSA in PBST for 1 h at room temperature then incubated with primary antibodies (Supplementary Table S2) overnight at 4 °C. The following day, membranes were washed three times with PBST and incubated with secondary antibodies for 1 h at room temperature. Finally, the bands were detected by chemiluminescence.

### Lentivirus infection for stable cells and plasmids transfection

Target cells were seeded in a tissue culture dish or plate at an appropriate density. The required volume of lentiviral particles was calculated based on the desired multiplicity of infection (MOI) and cell number. Lentiviral particles, along with polybrene at a final concentration of 10 µg/ml, were added to the target cells. The cells were incubated with the lentiviral particles for 12 h at 37 °C. Afterward, the medium was replaced with fresh growth medium, and the cells were cultured. After 48–72 h, transduced cells were selected with puromycin. Selection was monitored, and cells were passaged as needed while maintaining selection pressure. Once the selection was complete, the stable cell line was expanded, and validation experiments were performed.

### Cell proliferation and colony formation assays

ESCC cells were seeded at an appropriate density (2,000 cells/well) in a 96-well plate. Cells were cultured in the appropriate growth medium for the desired time period. Cell Counting Kit-8 (Cat# A311-02, Vazyme) was added to each well according to the manufacturer’s instructions. Plates were incubated for 1–2 h at 37 °C. The absorbance was measured at the appropriate wavelength using a plate reader. Cells were seeded at a density of 2,000 cells/well in a 6-well plate and cultured in the appropriate growth medium for 7–14 days, with medium changes every 3–4 days. The medium was aspirated, and cells were washed with PBS. Cells were fixed with methanol or 4% paraformaldehyde for 10 min, then stained with crystal violet for 30 min. The plate was rinsed with water to remove excess dye, air-dried, and the number of visible colonies was counted.

### MG132 and cycloheximide (CHX) treatment

ESCC cells were transfected with the indicated plasmids and treated with MG132 (10 µM, Cat# S2619, Selleck) or CHX (100 µg/mL, Cat# S7418, Selleck) for the indicated time points. Samples were collected in RIPA lysis buffer, fractionated by SDS-PAGE, and analyzed by WB.

### Immunoprecipitation

Cellular lysis was performed using IP lysis buffer (Cat# 87788, Thermo Fisher Scientific) containing protease inhibitor cocktail (Cat# 11836153001, Sigma-Aldrich) and phosphatase inhibitors (Cat# 4906845001, Sigma-Aldrich), with subsequent ice incubation for 15 min. Lysates were clarified by centrifugation at 16,000×g (4 °C, 15 min). Following pre-clearing with protein G agarose beads (Cat# 16–266, Sigma-Aldrich), 5 mg of lysate was immunoprecipitated with specified antibodies overnight at 4 °C. Immune complexes underwent three sequential washes with ice-cold lysis buffer. Input and immunoprecipitated samples were separated via SDS-PAGE and analyzed by immunoblotting using designated antibodies.

### In vivo ubiquitination assay

Ubiquitination analyses were performed as follows: HEK293T or ESCC cells were transiently transfected with HA-ubiquitin and other indicated plasmids using Lipofectamine 3000 reagent. Following 24-h transfection, cells were treated with MG132 (10 µM, Cat# S2619, Selleck) for 6 h in complete DMEM. Cellular lysates were prepared using IP lysis buffer and ultrasonicated for 5 min (10 pulses, 60 s each). The subsequent steps were the same as those for immunoprecipitation described above. Immunoprecipitates were then subjected to WB with the indicated antibodies.

### RNA stability assay

ESCC cells were plated at 5 × 10^4^ cells/well in 12-well plates and cultured. Cells were subjected to transcriptional arrest using 5 µg/mL actinomycin D (Cat# HY-17559, MedChemExpress) at indicated time points (0, 2, 4,8, 16 h post-treatment). The cells were harvested, and total RNA was extracted. The concentration and purity of the extracted RNA samples were determined using a spectrophotometer or fluorometric assay. RT-PCR was performed to quantify the levels of target RNA.

### PAR-CLIP

Photoactivatable-Ribonucleoside-Enhanced Crosslinking and Immunoprecipitation (PAR-CLIP) was performed following a standard protocol. Briefly, cells were grown in DMEM supplemented with 4-thiouridine (4-SU). Crosslinking was performed using a CL-1000 Ultraviolet Crosslinker (UVP) at 365 nm and 400 mJ/cm^2^. The supernatant was collected after centrifugation and digested using RNase T1, followed by the addition of calf intestinal alkaline phosphatase. The protein-RNA-bead complex was biotinylated using the RNA 3′ Terminal Biotinylation Kit (Cat# 20160, Thermo Fisher Scientific) according to manufacturer’s instructions. SDS-PAGE was performed to assess protein-RNA interactions. The relative density of RNA associated with the indicated protein was quantified.

### RNA immunoprecipitation (RIP) assay

RIP assay was performed using the Magna RIP™ RNA-Binding Protein Immunoprecipitation Kit (Cat# 17–700, Millipore) following the manufacturer’s protocol. Briefly, magnetic beads conjugated with 5 µg of species-matched control antibody were incubated with pre-cleared cell lysates at 4 °C for 16 h with gentle rotation. RNA-protein complexes were subsequently washed six times with kit-provided wash buffer and subjected to proteinase K (0.5 mg/mL) digestion in SDS-containing buffer. RNA was purified through acid phenol-chloroform extraction followed by ethanol precipitation. The relative interaction between NSUN2 or ALYREF and SREBP2 transcripts was determined by qPCR and normalized to the input.

### Cholesterol measurement assay

Cholesterol levels in the indicated cells were measured as previously described [31]. Briefly, cellular lipids were collected using chloroform:2-propanon: NP-40 (7:11:0.1) in a microhomogenizer, and cholesterol level were detected according to the manufacturer’s instructions using a Total Cholesterol Assay Kit (Cell Biolabs).

### Filipin III staining

A total of 3 × 10^4^ cultured cells were preseeded in 24-well plates. The following day, the indicated cells were fixed with 4% paraformaldehyde and treated with 0.05 mg/ml filipin III (Cat# HY-N6718, MedChemExpress) working solution for 2 h at room temperature.

### Seahorse bioanalyzer

60 min before the assay, cells were washed twice and transferred to fatty acid oxidation (FAO) assay medium composed of 111 mM NaCl, 4.7 mM KCl, 1.25 mM CaCl_2_, 2 mM MgSO_4_, and 1.2 mM NaH_2_PO_4_, supplemented with 2.5 mM glucose, 0.5 mM carnitine, and 5 mM HEPES (pH 7.4). Palmitate-BSA was added to a final concentration of 0.1 mM before assay initiation. FAO activity was assessed using the XFp Cell Mito Stress Test protocol. Drug concentrations during FAO measurements were as follows: oligomycin (Oligo), 4 µM; FCCP, 2 µM; antimycin A/rotenone (AA/Rot), 2 µM. FAO rates were normalized based on protein levels in each well.

### Flow cytometry analysis

Single-cell suspensions were resuspended in FACS buffer (PBS with 1% BSA) and blocked using anti-mouse CD16/32 antibodies for 10 min at room temperature. Cells were then stained with fluorochrome-conjugated antibodies against surface markers (BioLegend). Flow cytometry data were acquired on either a BD FACS Fortessa flow cytometer using BD FACSDiva software and analyzed using FlowJo software.

### Evaluation of m5C methylation

m5C methylation in the indicated cells was performed as previously described [32]. Briefly, 1 µg of anti-5-methylcytosine (m5C) antibody, 20 µg of total cellular RNA, and 20 µL of Protein G-conjugated agarose beads were co-incubated in 200 µL IPP buffer supplemented with 1 U/µL RNase inhibitor for 2 h at 4 °C with gentle agitation. After that, the beads were washed three times and RNA isolated from beads was analyzed by RT-PCR.

### Luciferase reporter gene assay

SREBP2 3’UTR reporter assays were performed as follows: The fragments of the SREBP2 3’UTR were subcloned into the downstream luciferase cassette of the pmirGLO dual-luciferase vector (Obio Technology, Shanghai, China). For functional analysis, 200 ng of pmirGLO constructs were co-transfected with 50 ng pRL-SV40 Renilla normalization vector (Cat# E2231, Promega) into ESCC cells (cultured in 24-well plates) using Lipofectamine 3000 reagent with parallel transfection groups. Luciferase activity and RNA expression were quantified at 48 h post-transfection using the Dual-Luciferase^®^ Reporter Assay System (Cat# E1910, Promega) and SYBR Green-based qRT-PCR, respectively, with firefly luciferase signals normalized to Renilla luciferase.

### Animal studies

Generation of *Ttpal-*knockout (KO) mice was achieved through CRISPR/Cas9-mediated genome editing. Briefly, Cas9 mRNA and two single-guide RNAs (sgRNAs) targeting exon 2–5 of *Ttpal* were microinjected into C57BL/6J zygotes. Genomic DNA extracted from tail biopsies was subjected to Sanger sequencing for genotype confirmation. Age-matched cohorts of 6-week-old wild type (WT) and *Ttpal-KO* mice (*n* = 15/group) were administered 100 µg/mL 4-nitroquinoline 1-oxide (4NQO, Cat# HY-33354, MedChemExpress) in drinking water for 16 weeks. Following 4NQO treatment, the mice were provided normal water until sacrificed. Esophageal tissue samples were collected for visual and histopathological evaluations of tumor development. Mice in the simvastatin treatment group received 3 mg/kg simvastatin via intraperitoneal injection three times weekly, whereas control animals were administered an isovolumetric vehicle formulation (2% DMSO, 30% polyethylene glycol 400 (PEG 400), and 5% Tween 80) under identical conditions.

For established in vivo tumorigenicity models, ESCC cells were subcutaneously inoculated into 6-week-old nude mice. Tumor nodules were excised and assessed for tumor volume and mass following a defined post-inoculation growth period. In dietary intervention experiments employing either a no cholesterol diet (Teklad Global 2018 C) or high cholesterol diet (Teklad Custom Diet TD.01383 containing 2% cholesterol), mice were maintained on their respective dietary regimens for 14 days prior to tumor cell implantation.

For the PDX models, Patient-derived ESCC tissues were detached from the adipose tissue, muscle layer, and connective tissue and obtained from the Shanghai General Hospital. This was followed by washing three times with PBS containing 1% penicillin/streptomycin and transported to a DMEM/F12 basal medium. Next, the tumor tissues were cut into 1- to 2-mm pieces and then implanted subcutaneously into female NCG mice. The ESCC tissues were divided into groups according to high or low TTPAL expression for the drug treatment assay. All experiments were reviewed and approved by the Animal Ethics Committee of Shanghai General Hospital, and the specimens were reviewed and approved by the hospital’s ethics committee.

### RNA-seq Library Preparation and Analysis

The RNA-Seq raw reads were processed using the standard filtering pipeline and subsequently aligned to the hg38 reference genome using HISAT2 (v2.1.0). Gene expression quantification was performed with HTSeq (v0.12.4) to enumerate reads mapped to annotated genes (GENCODE v25). Differential gene expression analysis was conducted using the DESeq2 package, with statistically significant changes defined as meeting dual criteria: absolute fold-change ≥ 1.5 and false discovery rate (FDR) ≤ 0.05.

### Public data processing

The expression data and CNV data of the TCGA cohort were downloaded from the cBioPortal website (https://www.cbioportal.org/), the UALCAN website (https://ualcan.path.uab.edu/index.html) and the GEPIA2 website (http://gepia2.cancer-pku.cn/#index).

### Statistical analysis

GraphPad Prism software (version 8.0, GraphPad, La Jolla, CA, USA) was used to perform statistical analysis, and the Kaplan–Meier method was used for survival analysis. Statistical significance was set at *p <* 0.05.

## Results

### TTPAL expression is elevated in ESCC due to copy number gain and associated with poor survival of patients with ESCC

**Fig. 1:**
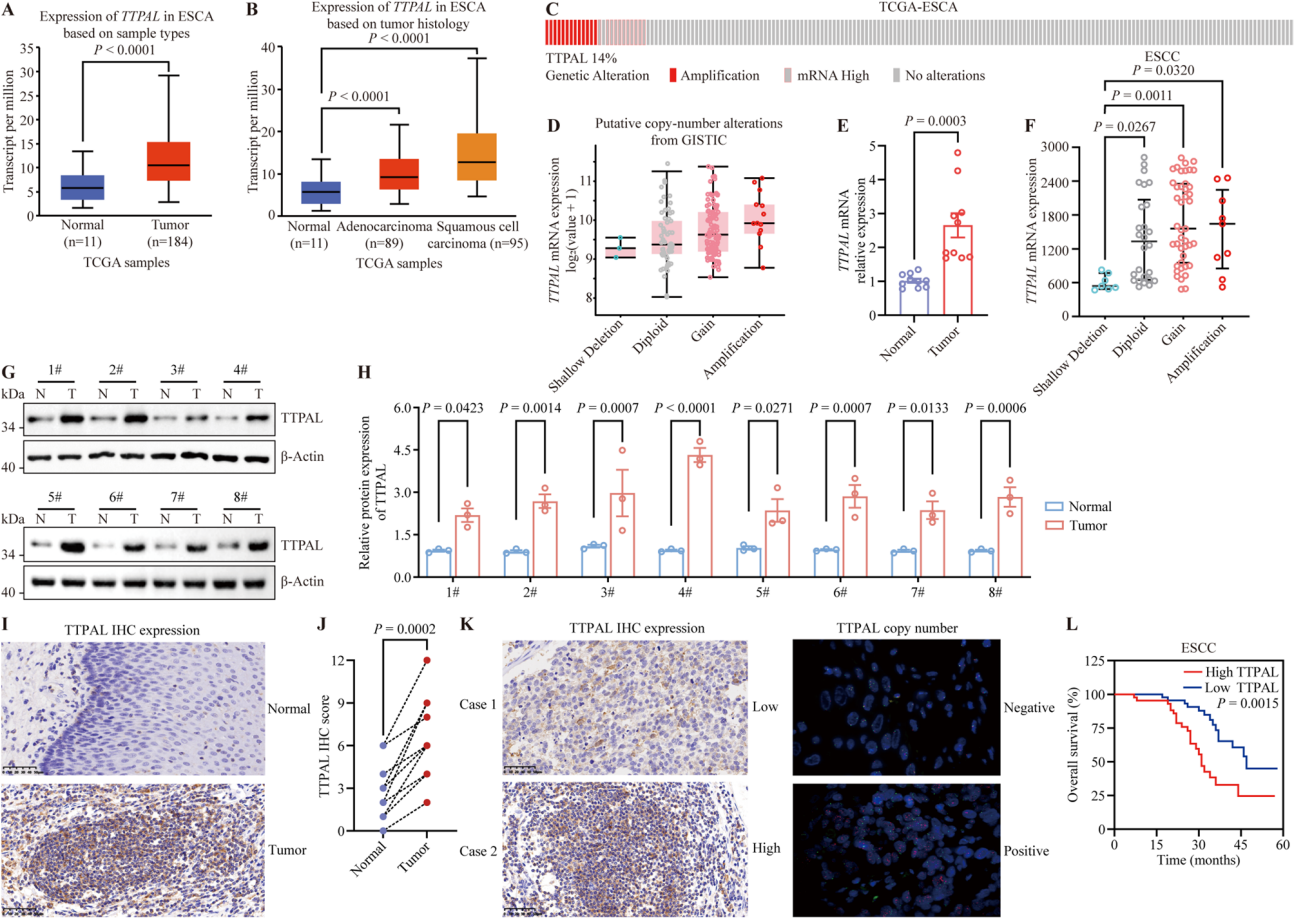
Elevated TTPAL expression associated with poor clinical outcomes in ESCC patients. **A***TTPAL* mRNA level of tumor tissues versus normal tissues in TCGA esophageal carcinoma cohort (https://ualcan.path.uab.edu/index.html). **B***TTPAL* mRNA level of esophageal adenocarcinoma, esophageal squamous cell carcinoma normal tissues in TCGA cohort (https://ualcan.path.uab.edu/index.html). **C** TCGA database in Cbioport website revealed mRNA expression and copy number alteration profile of *TTPAL*. **D***TTPAL* expression of tumor tissues with different *TTPAL* copy number in TCGA esophageal carcinoma cohort. **E***TTPAL* mRNA levels were significantly upregulated in tumor tissues compared with paired normal tissues in our ESCC cohort (*n* = 10). **F***TTPAL* expression of tumor tissues with different TTPAL copy number in our ESCC cohort. **G**,** H** WB analysis (**G**) and statistical quantification (**H**) revealed higher levels of TTPAL protein in tumor tissues compared with paired normal tissues in our ESCC cohort. (*n* = 8). **I**,** J** Representative IHC images (**I**) and IHC score (**J**) of TTPAL protein levels in tumors and paired normal tissues (*n* = 10). **K** Consistency between IHC and FISH results: Case 1 demonstrates low TTPAL expression and FISH negative; Case 2 exhibits high TTPAL expression and FISH positive **L** Kaplan-Meier analysis of the overall survival of ESCC patients in relation to different TTPAL expression levels

To identify the function of TTPAL in ESCC progression, we first found that *TTPAL* mRNA levels were significantly higher in tissues of patients with esophageal cancer than in normal tissues in TCGA cohort (Fig. 1A and B). Interestingly, *TTPAL* mRNA expression positively correlated with DNA copy number in TCGA ESCC cohort (Fig. 1C and D). To further verify the relationship between *TTPAL* mRNA expression and DNA copy number, we confirmed that *TTPAL* mRNA expression was upregulated and positively correlated with the DNA copy number in the ESCC cohort (Fig. 1E and F). We also evaluated the TTPAL protein levels in ESCC tissues. TTPAL protein levels were higher in ESCC tissues than in the adjacent normal tissues (Fig. 1G-J). To investigate the relationship between *TTPAL* DNA amplification and its overexpression, *TTPAL* DNA copy number alterations were analyzed by FISH comprising 88 ESCC cases (Fig. 1K). Table 1 presents the concordance rate of TTPAL status assessed by IHC and FISH assays. Among the 32 cases with TTPAL high expression (IHC 3+), 78.12% (25/32) showed concordance between copy number amplification and protein expression levels. In the 22 patient samples without TTPAL high expression (IHC 0/1+), approximately 90% (20/22) cases without *TTPAL* copy number gain (Table 1). Although the Kappa value was 0.353, likely due to the limited number of cases collected in this study. These findings indicate that the upregulation of TTPAL protein expression in ESCC is partially attributable to DNA amplification events. Kaplan‒Meier survival curves showed that ESCC patients with high TTPAL expression had worse overall survival than patients harboring low-TTPAL levels (Fig. 1L). All the above results show that *TTPAL* is amplified in the 20q13.12 region and is highly expressed in ESCC patients, with clinical prognostic value.

**Table 1:** Comparison of HER-2 results determined by IHC and FISH

IHC	FISH		Concordance by IHC	Discordance by IHC
	positive	negative		
3+ (*n* = 32)	25	7	(25/32)78.12%	(7/32)21.88%
2+ (*n* = 34)	30	4	(30/34)88.24%	(4/34) 11.76%
0/1+ (n = 22)	2	20	(20/22)90.91%	(2/22)9.09%

Κappa = 0.353, *p* < 0.01

### TTPAL promotes tumorigenesis and progression of ESCC

**Fig. 2:**
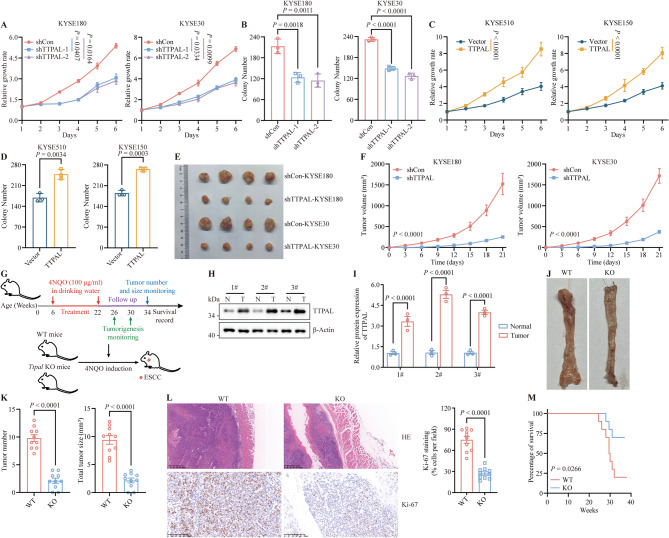
TTPAL promotes ESCC cell growth. **A** The growth curves of KYSE180 and KYSE30 cells with control or *TTPAL* depletion. **B** Cell colony formation of KYSE180 and KYSE30 cells with control or *TTPAL* depletion. **C** The growth curves of KYSE510 and KYSE150 cells with control or *TTPAL* overexpression. **D** Cell colony formation of KYSE510 and KYSE150 cells with control or *TTPAL* overexpression. **E**,** F** Photograph, and quantification of excised subcutaneous tumors harvested by injecting shNC versus shTTPAL in KYSE180 and KYSE30 cells. **G** A schematic diagram of timeline of constructing the 4NQO-induced tumorigenesis mouse model. **H**,** I** WB analysis (**H**) and statistical quantification (**I**) measured TTPAL levels in normal esophagus and esophageal cancer tissues from WT mice. **J** Representative images of the esophagus in 4NQO-induced WT and *Ttpal-*KO mice. **K** Tumor number (left) and tumor size (right) of esophagus in 4NQO-induced WT and *Ttpal-*KO mice. **L** Representative images of H&E-stained esophageal sections demonstrating morphological alterations (Top), representative images of Ki-67 immunohistochemical (Down) and analysis (Right) revealing epithelial cell proliferation patterns. **M** The overall survival of 4NQO-induced WT and *Ttpal-*KO mice

To explore the relationship between TTPAL and ESCC progression, we measured TTPAL protein levels in ESCC cells and selected four cell lines (KYSE180, KYSE30, KYSE510, and KYSE150) with different TTPAL expression levels for further experiments (Supplementary Fig. 1A, 1B). We then knocked down *TTPAL* in KYSE180 and KYSE30 cells, overexpressed *TTPAL* in KYSE510 and KYSE150 cells, and confirmed the efficacy of *TTPAL* knockdown and overexpression by WB (Supplementary Fig. 1C-E). In vitro loss- and gain-of-function assays were performed to investigate the role of *TTPAL* in ESCC progression. Knockdown of *TTPAL* using shRNA in KYSE180 and KYSE30 cells suppressed their proliferation and clonogenicity (Fig. 2A and B and Supplementary Fig. 1F). Consistent with these results, the overexpression of *TTPAL* in cells remarkably enhanced cell proliferation and clonogenicity (Fig. 2C and D and Supplementary Fig. 1G).

Based on these in vitro findings, we used subcutaneous xenograft mouse models and found that *TTPAL* knockdown in ESCC cells markedly inhibited the increase of tumor volume over the entire assay period, leading to a decrease in the final tumor weight (Fig. 2E and F and Supplementary Fig. 1H). Inoculation of control and *TTPAL* knockdown ESCC cells subcutaneously showed no significant impact on nude mice body weight (Supplementary Fig. 1I). We generated *Ttpal*-knockout (KO) mice to verify the oncogenic role of *Ttpal* in vivo. The body size and organs of *Ttpal*-KO mice were not significantly different from those of WT mice (Supplementary Fig. 1J). The differentiation of bone marrow cells from *Ttpal*-KO mice into BMDCs and BMDMs in GM-CSF and M-CSF cultures, respectively, was similar to that of WT cells (Supplementary Fig. 1K). Furthermore, cytometry analysis revealed comparable immune cell numbers and composition in the spleen and peripheral lymph nodes of WT and *Ttpal*-KO mice (Supplementary Fig. 1L, M). These findings indicate that *Ttpal* knockout does not affect immune cell homeostasis. Then, we used the chemical carcinogen 4-nitroquilonince *N*-oxide (4NQO) to induce ESCC in WT and KO mice (Fig. 2G). We observed atypical esophageal hyperplastic lesions or ESCC in WT mice after 4NQO withdrawal for approximately 4–8 weeks (Supplementary Fig. 2A). WB assays showed Ttpal protein expression was upregulated in ESCC tumor tissues compared with normal esophageal epithelium (Fig. 2H and I). Relative to those of WT mice, we observed that both the number and size of tumor in *Ttpal*-KO mice were reduced (Fig. 2J and K). Histopathological evaluation and Ki-67 staining also revealed that WT mice exhibited more advanced esophageal tumor stages than *Ttpal*-KO mice following 4NQO withdrawal for 12 weeks (Fig. 2L). IHC analysis revealed no significant differences in CD3 + T cell, CD19 + B cell, or F4/80 + macrophage density between esophageal cancer tissues from WT and *Ttpal-*KO mice (Supplementary Fig. 2B). Consistent with these results, *Ttpal*-KO mice had a better prognosis than WT mice (Fig. 2M). These results demonstrated that TTPAL plays an oncogenic role in ESCC progression.

### TTPAL potentiates cholesterol biosynthesis in ESCC

**Fig. 3:**
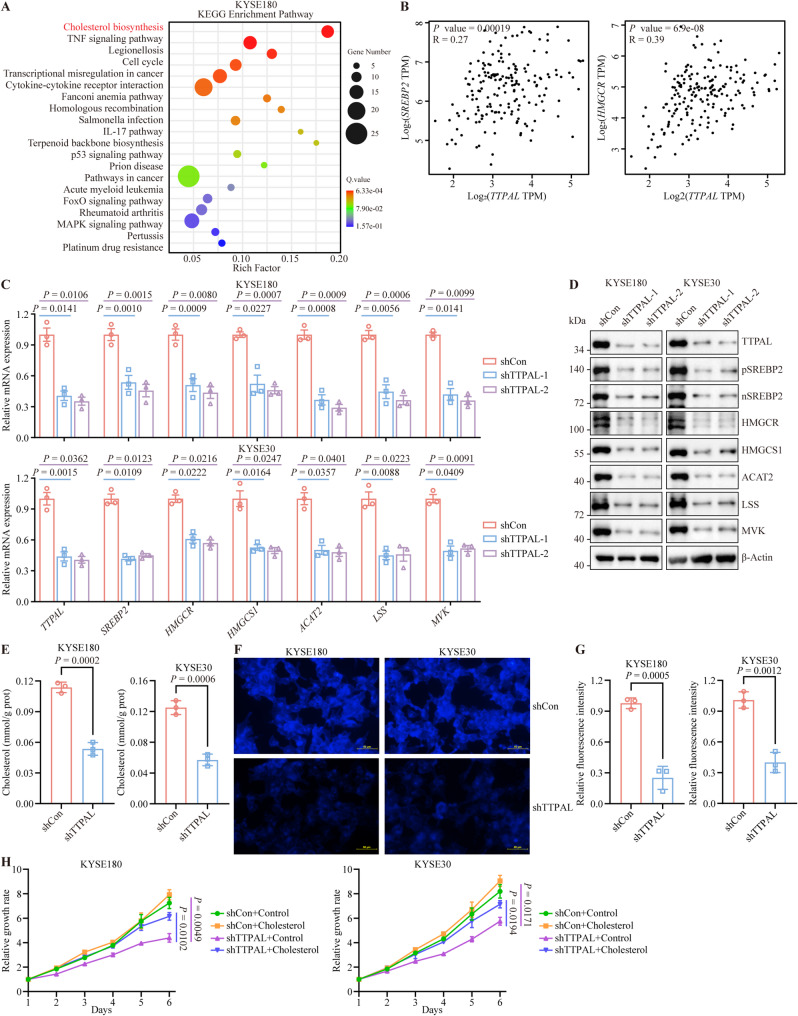
TTPAL plays an important role in cholesterol biosynthesis in ESCC. **A** KEGG pathways enriched by differently expressed genes influenced by *TTPAL* in KYSE180. **B** The correlated expression of *TTPAL* and *SREBP2*, *HMGCR* in TCGA-ESCA cohorts in GEPIA2. **C** RT-PCR analysis of mRNA levels of cholesterol biosynthesis pathway genes after *TTPAL* depletion in KYSE180 and KYSE30 cells. **D** WB analysis of cholesterol biosynthesis pathway genes after *TTPAL* depletion in KYSE180 and KYSE30 cells. **E** Levels of total cholesterol in KYSE180 and KYSE30 cells with *TTPAL* depletion. **F**,** G** Filipin III staining (**F**) and statistical quantification (**G**) displaying the cellular total cholesterol level in KYSE180 and KYSE30 cells with *TTPAL* depletion. **H** CCK-8 assay revealing that cholesterol treatment rescued the inhibitory effect of *TTPAL* knockdown on the growth ability of KYSE180 and KYSE30 cells

To explore the mechanism of action of TTPAL in ESCC cells, we performed RNA sequencing of *TTPAL*-depleted and control KYSE180 cells. Kyoto Encyclopedia of Genes and Genomes (KEGG) pathway enrichment analysis showed that cholesterol biosynthesis was the most enriched pathway in *TTPAL*-silenced cells (Fig. 3A). Interestingly, we also observed that *TTPAL* was significantly associated with cholesterol metabolism in a genome wide association study (GWAS) (https://www.genecards.org/cgi-bin/carddisp.pl?gene=TTPAL). In TCGA dataset, the expression of *TTPAL* mRNA was positively correlated with the expression levels of *SREBP2* mRNA and its downstream target genes in different types of cancers (Fig. 3B and Supplementary Fig. 3). These results prompted us to investigate the role of *TTPAL* in cholesterol biosynthesis. We further confirmed that the mRNA and protein levels of full and cleaved SREBP2 and its downstream target genes decreased following *TTPAL* silencing in ESCC cells (Fig. 3C and D and Supplementary Fig. 4A). *TTPAL* knockdown caused a remarkable decrease in cholesterol levels in ESCC cells (Fig. 3E-G). We also evaluated fatty acid oxidation (FAO) in *TTPAL*-deficient ESCC cells using the Seahorse XFp Cellular Flux Analyzer. *TTPAL* depletion did not significantly affect FAO in ESCC cells (Supplementary Fig. 4B).

To investigate the potential inhibitory effect of cholesterol reduction on ESCC proliferation, we performed comparative analyses of cell growth in media supplemented with normal fetal bovine serum versus lipoprotein-depleted FBS (LPDS). Our results demonstrated that ESCC cells cultured in delipidated medium exhibited significantly attenuated proliferative capacity compared to controls. Notably, supplementation of LPDS medium with 5 µM cholesterol effectively rescued cellular proliferation rates to levels comparable with those observed in normal FBS culture conditions (Supplementary Fig. 5A). Furthermore, we observed that cholesterol treatment restored the proliferation of *TTPAL*-silenced cells (Fig. 3H). To evaluate the tumor-promoting effects of dietary cholesterol on ESCC, we established xenograft models by subcutaneously implanting ESCC cell lines into nude mice. Animals were randomized into two dietary regimens: a cholesterol-free control diet (NC, 0% cholesterol) or a hypercholesterolemic diet (HC, 2% cholesterol). Notably, HC-fed mice exhibited significantly accelerated tumor progression compared to NC diet controls (Supplementary Fig. 5B). These findings collectively establish that TTPAL promotes ESCC cell proliferation and tumor growth through enhanced cholesterol biosynthesis.

### TTPAL binds with and stabilizes NSUN2 protein to increase SREBP2 expression

**Fig. 4:**
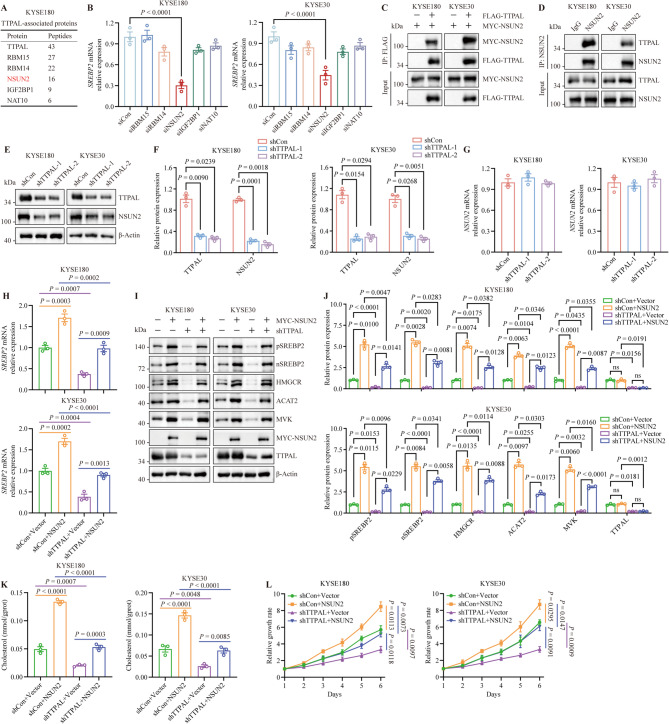
TTPAL interacts with NSUN2 to activate cholesterol biosynthesis in ESCC cells. **A**. The proteins binding to TTPAL in HEK293T cells examined by coimmunoprecipitation coupled with mass spectrometry (CoIP-MS). **B** RT-PCR showed the *SREBP2* mRNA expression after knocking down the indicated genes by their corresponding siRNA. **C** KYSE180 and KYSE30 cells were transfected with FLAG-TTPAL, MYC-NSUN2 as indicated. CoIP and WB were conducted to prove the interaction between FLAG-TTPAL and MYC-NSUN2. **D** CoIP and WB were conducted to prove the interaction between TTPAL and NSUN2 in ESCC cells. **E**,** F** WB analysis (**E**) and statistical quantification (**F**) measured the NSUN2 expression levels in KYSE180 and KYSE30 cells with TTPAL depletion. **G** RT-PCR measured the *NSUN2* mRNA expression levels in KYSE180 and KYSE30 cells with *TTPAL* depletion. **H-J** RT-PCR (**H**) and WB (**I**,** J**) revealing that overexpression of *NSUN2* rescued the inhibitory effect of *TTPAL* knockdown on the expression levels of cholesterol biosynthesis pathway in KYSE180 and KYSE30 cells. **K** Overexpression of *NSUN2* rescued the inhibitory effect of *TTPAL* knockdown on the cholesterol contents in KYSE180 and KYSE30 cells. **L** CCK-8 assay revealed that overexpression of *NSUN2* rescued the inhibitory effect of *TTPAL* knockdown on the growth ability of KYSE180 and KYSE30 cells

To identify the mechanisms of cholesterol biosynthesis in ESCC cells, we performed immunoprecipitation-mass spectrometry analysis and found that TTPAL interacts with a range of RNA-binding proteins, including RBM15, RBM14, NSUN2, IGF2BP1, and NAT10 (Fig. 4A). Previous studies have shown that RNA-binding proteins regulate the stability or efficacy of targets by increasing or decreasing their expression levels. To determine whether TTPAL regulates SREBP2 expression via RNA-binding proteins, we knocked down various RNA-binding proteins in ESCC cells. Only *NSUN2* silencing reduced *SREBP2* expression in ESCC cells (Fig. 4B). Next, we examined the interaction between TTPAL and NSUN2. We performed a co-immunoprecipitation-western blot assay for FLAG-TTPAL and MYC-NSUN2 expression in ESCC cells and found a strong association between TTPAL and NSUN2 (Fig. 4C). Consistent with this result, an interaction between endogenous TTPAL and NSUN2 was observed in ESCC cells (Fig. 4D). We also found that NSUN2 expression levels were decreased in *TTPAL*-depleted ESCC cells. *TTPAL*-overexpression increased NSUN2, pSREBP2 and nSREBP2 in ESCC cells; however, *NSUN2* mRNA levels did not change significantly in *TTPAL*-depleted ESCC cells (Fig. 4E-G and Supplementary Fig. 6A, 6B).

Next, we evaluated whether regulation of SREBP2 expression by TTPAL was dependent on NSUN2. We overexpressed *NSUN2* in *TTPAL*-silenced ESCC cells and found that the expression of SREBP2 and its downstream cholesterol biosynthesis enzymes was restored by *NSUN2* overexpression (Fig. 4H-J). Analysis of TCGA-ESCA data on GEPIA showed a significant positive correlation between *NSUN2* and *SREBP2* expression (Supplementary Fig. 6C). The protein expression levels of TTPAL, SREBP2, and NSUN2 were measured in out cohorts. Representative IHC images are shown in Supplementary Fig. 6D, and the IHC results showed that TTPAL expression positively correlated with SREBP2 and NSUN2 expression (Supplementary Fig. 6E). Furthermore, the cholesterol levels were restored by *NSUN2* overexpression in *TTPAL*-depleted ESCC cells (Fig. 4K). Consistent with these results, the proliferation of *TTPAL*-silenced cells was restored following *NSUN2* overexpression (Fig. 4L). Taken together, these data suggest that TTPAL-NSUN2 signaling plays a crucial role in cholesterol biosynthesis to promote ESCC progression.

### TTPAL stabilizes NSUN2 protein by interrupting STUB1-mediated ubiquitination degradation

**Fig. 5:**
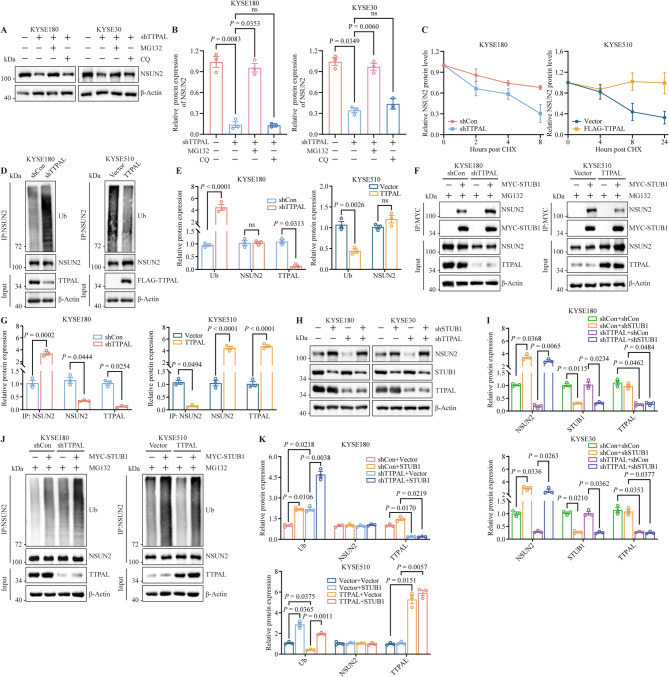
TTPAL stabilizes NSUN2 by interrupting STUB1-mediated ubiquitination degradation of NSUN2 in ESCC cells. **A, B** WB analysis (**A**) and statistical quantification (**B**) of NSUN2 expression in KYSE180 cells and KYSE30 cells treated with MG132 (10 µM, 6 h) or CQ (100 nM, 12 h). **C** NSUN2 expression levels in ESCC cells treated with cycloheximide (CHX) for the indicated time points. **D**,** E** WB analysis (**D**) and statistical quantification (**E)** measured ubiquitination of NSUN2 in *TTPAL* depletion or *TTPAL* overexpression ESCC cells. **F**,** G** CoIP analyses (**F**) and statistical quantification (**G**) displaying that the interaction of STUB1 and NSUN2 in ESCC cells with *TTPAL* knockdown or overexpression ESCC cells. **H**,** I** WB analysis (**H**) and statistical quantification (**I**) measured NSUN2 expression in *STUB1* knockdown with or without *TTPAL* knockdown. **J**,** K** WB analysis (**J**) and statistical quantification (**K**) measured ubiquitination of NSUN2 in *TTPAL* depletion or *TTPAL* overexpression ESCC cells with or without transfected MYC-STUB1

Since there are two main protein degradation pathways (proteasomes and lysosomes) in eukaryotic cells, we explored the specific mechanism using the proteasome inhibitor MG132 and the lysosome inhibitor bafilomycin A1 (Chloroquine, CQ). The results showed that only MG132 reversed the *TTPAL*-depletion-induced decrease in NSUN2 expression levels, demonstrating that TTPAL regulates NSUN2 expression levels in a proteasome-dependent manner (Fig. 5A and B). Furthermore, TTPAL stabilized NSUN2 after treatment with the protein synthesis inhibitor cycloheximide (CHX) (Fig. 5C and Supplementary Fig. 7A, 7B). Consistent with these results, NSUN2 ubiquitination was upregulated by *TTPAL* knockdown and downregulated by *TTPAL* overexpression in ESCC cells (Fig. 5D and E).

Given the ability of TTPAL to directly inhibit NSUN2 ubiquitination, we speculated that a deubiquitinase or E3 ligase might regulate TTPAL-mediated NSUN2 expression. A previous study reported that STUB1 modifies the ubiquitination of NSUN2 at lysines 457 and 654, resulting in NSUN2 degradation [33]. Thus, we first found in ESCC cells the interaction between endogenous STUB1 and NSUN2 in using immunoprecipitation-western blotting (Supplementary Fig. 7C). We also observed that *TTPAL* knockdown in ESCC cells enhanced the interaction between STUB1 and NSUN2, and STUB1-mediated ubiquitination. In contrast, the STUB1-NSUN2 interaction was markedly weakened when *TTPAL* was overexpressed (Fig. 5F and G). *STUB1* silencing restored NSUN2 protein levels in *TTPAL*-depleted ESCC cells (Fig. 5H and I). We explored whether TTPAL regulates the ubiquitination of NSUN2 by affecting NSUN2 binding to STUB1. Our results revealed that *TTPAL* knockdown promotes STUB1-mediated NSUN2 ubiquitination. Consistent with these observations, NSUN2 ubiquitination was downregulated upon *TTPAL* overexpression (Fig. 5J and K). Taken together, these data demonstrate that TTPAL stabilizes NSUN2 by inhibiting the STUB1-mediated ubiquitination and degradation of NSUN2.

### NSUN2 promotes SREBP2 expression via regulating the m5C modification of *SREBP2* mRNA

**Fig. 6:**
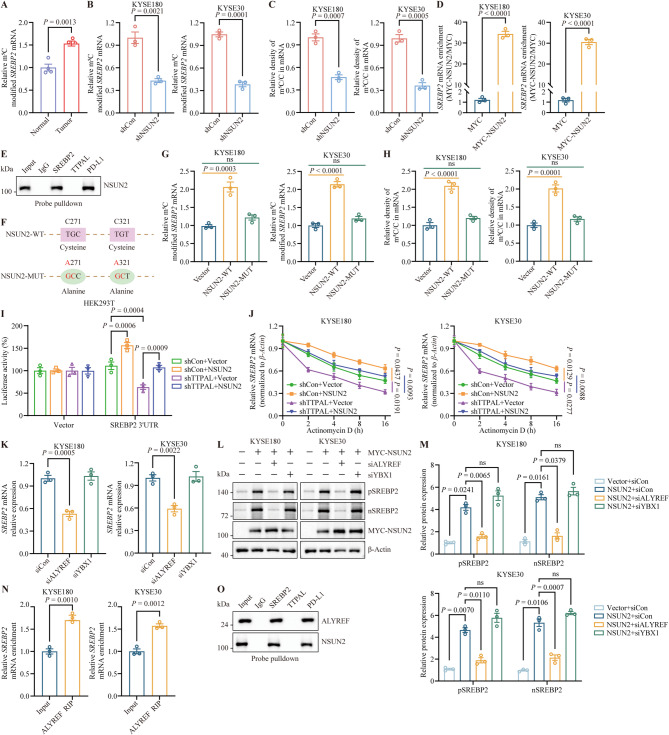
NSUN2 increased SREBP2 expression and cholesterol biosynthesis by upregulating the m5C modification of *SREBP2* mRNA. **A** RT-PCR detected the level of m5C-modified *SREBP2* mRNA in the ESCC patients’ tumor tissues and their adjacent non-tumor tissues. **B** RT-PCR showed the level of m5C-modified *SREBP2* mRNA in the KYSE180 and KYSE30 with control or NSUN2 depletion. **C** The m5C level of total mRNA in the KYSE180 and KYSE30 with control or *NSUN2* depletion. **D** RIP assay showed that the NSUN2 interacted with *SREBP2* mRNA. **E** Biotinylated probe recognizing *SREBP2*, *PD-L1* and *TTPAL* mRNA pulled down lysates of KYSE180, and the WB detected NSUN2 in the precipitates. **F** The mutant *NSUN2* plasmid contains two-point mutations including catalytic site (C321A) and releasing (C271A). **G** RT-PCR assay detected the level of m5C-modified *SREBP2* mRNA in the KYSE180 and KYSE30 with control, wild-type *NSUN2* plasmids and *NSUN2* plasmid with point mutations (C271A and C321A) overexpression. **H** The m5C level of total mRNA was detected in the KYSE180 and KYSE30 cells transfected with control, wild-type *NSUN2* plasmids and *NSUN2* plasmid with point mutations (C271A and C321A) overexpression. **I** The psiCHECK^TM^-2 containing *SREBP2* 3’UTR was co-transfected into HEK293T cells with shTTPAL or *NSUN2*, along or together co-transfected, then luciferase reporter assays measured the luciferase activities of *SREBP2* 3’UTR luciferase reporter. **J** The indicated plasmids were transfected into KYSE180 and KYSE30 cells, and then treated with actinomycin D at different time points. RT-PCR detected the stability of *SREBP2* mRNA. **K** KYSE180 and KYSE30 cells were transfected with indicated siRNA and the RT-PCR determine the *SREBP2* mRNA expression level. **L**,** M** WB assay (**L**) and statistical quantification (**M**) measured the SREBP2 expression level in the KYSE180 and KYSE30 transfected with *NSUN2* and the specific siRNA. **N** RIP assay showed that the ALYREF interacted with *SREBP2* mRNA. **O** Biotinylated probe recognizing *SREBP2*, *PD-L1* and *TTPAL* mRNA pulled down lysates of KYSE180, and the WB detected NSUN2 and ALYREF in the precipitates

NSUN2 increases the expression of target genes by promoting m5C modification in various cancers. To examine whether the regulation of SREBP2 expression by NSUN2 is dependent on m5C modification of mRNA, we compared m5C modification levels in ESCC and normal tissues. As shown in Fig. 6A, the m5C modification of *SREBP2* was higher in tumor tissues than in normal tissues. We measured the m5C modification levels in both *NSUN2*-silenced and control ESCC cells (Fig. 6B). As expected, m5C modification was decreased in *NSUN2*-depleted ESCC cells compared to control cells (Fig. 6C). Consistent with these results, the m5C modification level of *SREBP2* mRNA was significantly upregulated in ESCC cells with *NSUN2* overexpression compared to that in control ESCC cells (Fig. 6D). To confirm whether NSUN2 directly binds to *SREBP2* mRNA, we observed that *SREBP2* mRNA was enriched in immunoprecipitates pulled-down using anti-MYC in MYC-NSUN2 ESCC cells (Fig. 6E). Overexpression of wild-type *NSUN2*, but not its mutant, increased m5C *SREBP2* mRNA levels and the proportion of m5C-modified mRNA within the total mRNA in ESCC cells (Fig. 6F-H, and Supplementary Fig. 8A, 8B). We also observed that the overexpression of *NSUN2*, but not its mutant, significantly upregulated SREBP2 protein and cholesterol levels in ESCC cells (Supplementary Fig. 8C). We then detected the luciferase activity by fused a luciferase reported to *SREBP2*-3’UTR. The results showed that *NSUN2* overexpression increased luciferase activity, whereas *TTPAL* knockdown inhibited luciferase activity and reversed the inhibitory effect of TTPAL on *NSUN2* overexpression (Fig. 6I). We then explored whether m5C modification catalyzed by *NSUN2* could stabilize *SREBP2* mRNA and performed RNA decay assays. The results showed that *NSUN2* overexpression increased *SREBP2* mRNA stability, whereas *TTPAL* knockdown decreased *SREBP2* mRNA stability and reversed the inhibitory effect of *TTPAL* on *NSUN2* overexpression (Fig. 6J). These results demonstrated that NSUN2 elevates SREBP2 expression by directly interacting with and modifying m5C *SREBP2* mRNA.

Given the ability of YBX1 and ALYREF to recognize and stabilize target m5C-modified mRNA, we examined whether YBX1 and/or ALYREF regulate SREBP2 expression. As shown in results, we found that only siALYREF, but not siYBX1, reduced *SREBP2* mRNA and protein levels in ESCC cells (Fig. 6K-M). Next, RNA-immunoprecipitation-PCR showed that *SREBP2* mRNA was remarkably enriched in the ALYREF antibody in the anti-ALYREF immunoprecipitates (Fig. 6N). Consistent with these results, a biotinylated probe binding to *SREBP2* mRNA, but not to *TTPAL* mRNA, pulled down the NSUN2 and ALYREF proteins (Fig. 6O). Collectively, these data demonstrate that ALYREF directly recognizes and stabilizes the m5C-modified *SREBP2* mRNA.

### TTPAL promotes therapeutic sensitivity to simvastatin

**Fig. 7:**
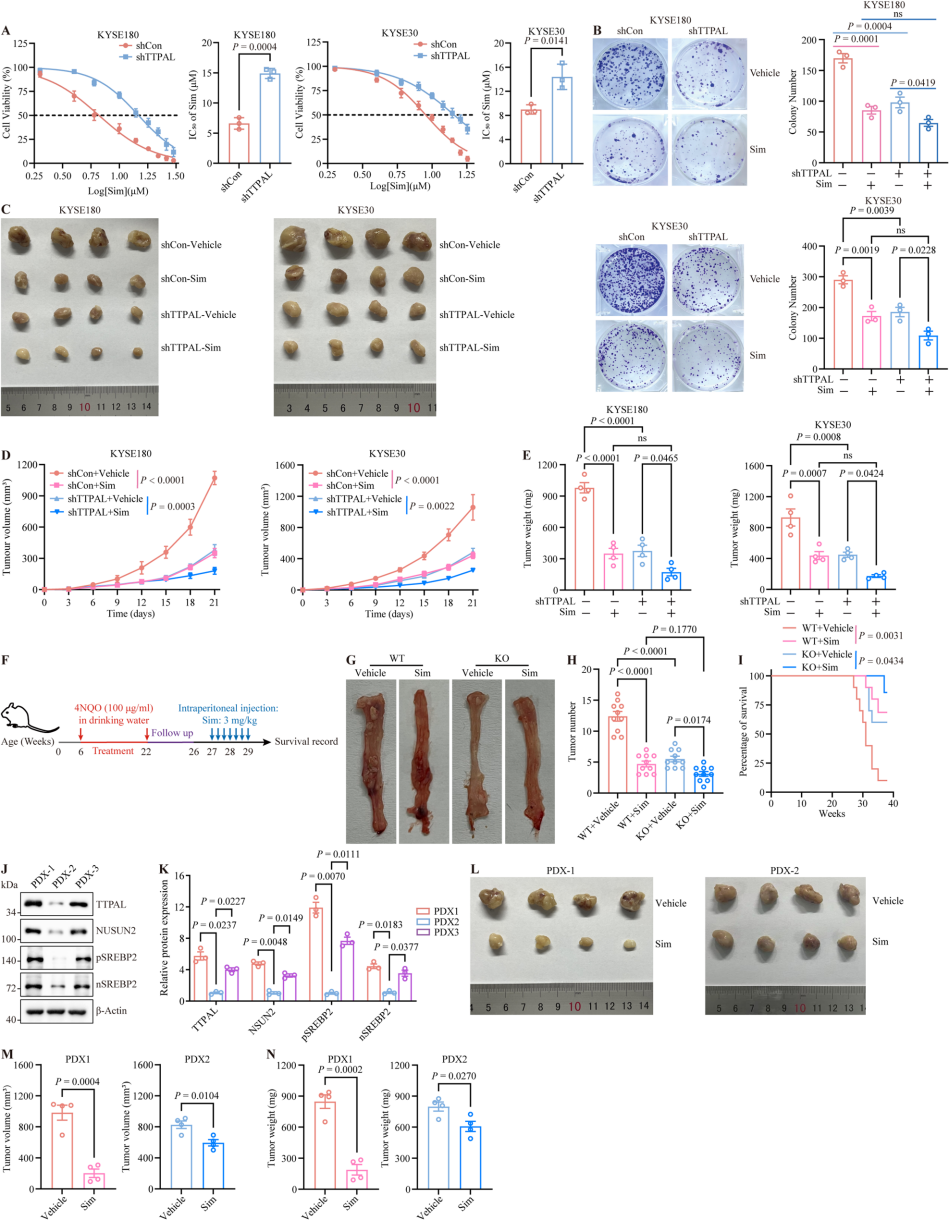
TTPAL enhances simvastatin sensitivity in ESCC cells. **A** Dose-response and IC_50_ values of simvastatin in KYSE180 cells and KYSE30 with control or *TTPAL* depletion. **B** Colony formation assay was performed to examine the effects of simvastatin on the growth ability of KYSE180 and KYSE30 cells with control or *TTPAL* depletion. **C-E** Tumor photographs (**C**), tumor growth kinetics (**D**), and tumor weight (**E**) measurements from nude mice harboring subcutaneous xenografts of KYSE180 (control and *TTPAL*-knockdown) or KYSE30 (control and *TTPAL*-knockdown) esophageal cancer cells treated with or without simvastatin. **F** A schematic diagram demonstrates 4NQO-induced carcinogenesis in the simvastatin treated mouse model. **G** The representative images of the esophagus in 4NQO-induced WT or *Ttpal-*KO mice treated with or without simvastatin. **H** Tumor number in esophagus of 4NQO-induced WT and *Ttpal-*KO mice treated with or without simvastatin. **I** The overall survival of 4NQO-induced WT or *Ttpal-*KO mice treated with or without simvastatin. **J**,** K** WB analysis (**J**) and statistical quantification (**K**) of TTPAL, SREBP2, and NSUN2 in three ESCC patients’ tumor tissues. **L-N** The tumor images (**L**), volume (**M**), and weight (**N**) of the PDX-1 and PDX-2 treated with or without simvastatin

Given the ability of TTPAL to activate cholesterol biosynthesis, we explored whether TTPAL could serve as a novel therapeutic target for the inhibition of cholesterol biosynthesis in ESCC cells. Simvastatin are small molecular inhibitors of cholesterol biosynthesis. Compared with the *TTPAL*-depleted group, the control group was more sensitive to simvastatin and displayed suppressed growth upon treatment with simvastatin (Fig. 7A and B). In contrast, *TTPAL* overexpression weakened the sensitivity of ESCC cells to simvastatin (Supplementary Fig. 9A). We also observed that simvastatin exhibited no significant alteration in intracellular cholesterol levels in *TTPAL*-knockdown ESCC cells, while also showing no notable impact on the expression of NSUN2 or simvastatin’s target HMGCR (Supplementary Fig. 9B-D). Consistent with in vitro results, simvastatin treatment showed better therapeutic efficacy in nude mice bearing subcutaneous esophageal cancer xenografts without *TTPAL* knockdown compared to those with *TTPAL* knockdown (Fig. 7C-E). To evaluate the effects of simvastatin on ESCC tumors in an immunocompetent background, we treated 4NQO-induced WT and *Ttpal*-KO mice with simvastatin. Simvastatin exhibited better therapeutic effects on 4NQO-induced esophageal cancer in WT mice compared with *Ttpal*-KO mice (Fig. 7F-H). In addition, compared with *Ttpal*-KO mice, simvastatin provided better survival benefits for WT mice bearing 4NQO-induced esophageal cancer (Fig. 7I). To evaluate the overall safety profile of simvastatin, we measured the major blood biochemical and cell parameters. The results suggest that simvastatin did not cause any significant impairment of complete cell count or function of liver and kidney (Supplementary Fig. 10A-C). There were no significant differences in body size and organs of mice treated with or without simvastatin (Supplementary Fig. 10D, 10E). These results collectively indicate that simvastatin administration is associated with minimal adverse effects in mice. To further elucidate therapeutic responses in patients with ESCC, we established ESCC-derived xenograft (PDX) models and quantified simvastatin efficacy relative to vehicle control. SREBP2 and NSUN2 expression levels exhibited significant positive correlations with TTPAL tumor expression (Fig. 7J and K). Furthermore, simvastatin demonstrated antitumor activity across both PDX1 and PDX2 models, although the therapeutic efficacy was less pronounced compared to PDX1, likely due to the relatively lower expression of TTPAL in PDX1 (Fig. 7L-N). In line with our in vitro observations, simvastatin exhibited no notable alteration in NSUN2 and HMGCR expression in PDX-1-derived tumor specimens (Supplementary Fig. 10F, 10G). Collectively, these findings indicate that TTPAL expression may serve as a novel biomarker for stratifying patients likely to benefit from cholesterol biosynthesis-targeted therapeutic strategies in ESCC.

## Discussion

Emerging evidence highlights the critical role of chromosomal numerical aberrations in oncogenesis by modulating oncogene and tumor suppressor expression [34, 35]. Current clinical in ESCC targeted therapy primarily focus on oncogenic drivers arising from copy number variations, notably *EGFR* (7p11.2) and *VEGFR* [36, 37]. As a proto-oncogenic transmembrane receptor tyrosine kinase belonging to the ErbB family, amplified *EGFR* activates canonical oncogenic signaling through both the RAS-MAPK (RAF-MEK-ERK) cascade and PI3K-AKT-mTOR axis [38]. While these established targets remain therapeutic mainstays, our study identifies *TTPAL* (20q13.12) as a novel candidate genomic amplification event in ESCC pathogenesis. Unlike EGFR’s canonical kinase-dependent signaling, *TTPAL* appears to exert oncogenic effects through cholesterol metabolic reprogramming via *SREBP2* upregulation. These functional results suggest *TTPAL* amplification represents a therapeutic axis in ESCC, potentially expanding the druggable landscape beyond conventional growth factor signaling targets.

Originally characterized as a putative phosphatidylinositol bisphosphate-binding protein, TTPAL has recently been implicated in cancer biology through distinct mechanisms [16, 17]. Initial reports identified its role in colorectal carcinogenesis through TRIP6 stabilization-mediated potentiation of Wnt/β-catenin signaling [17]. Subsequent mechanistic studies revealed TTPAL’s capacity to drive gastric cancer progression via AKT pathway activation [16]. Despite these advances, the metabolic regulatory functions of TTPAL across malignancies remain undefined. Our investigation establishes that *TTPAL* expression in ESCC directly correlates with its genomic amplification status, with significant overexpression observed in primary tumor specimens relative to matched normal epithelium. Through development of isogenic *TTPAL* knockdown models, we demonstrated marked proliferation deficits in ESCC cells. Complementary in vivo studies using a *Ttpal* knockout mouse model revealed that genetic ablation significantly attenuates 4-NQO-induced ESCC carcinogenesis. These collective findings demonstrate that *TTPAL* amplification and consequent overexpression might be as potentially initiating events in ESCC pathogenesis.

Interestingly, cholesterol biosynthesis was the most enriched pathway. Genome-wide association studies (GWAS) have also identified *TTPAL* to be associated with cholesterol metabolism. These results prompted us to study the regulatory role of *TTPAL* in cholesterol biosynthesis in ESCC cells. Cholesterol is a vital metabolite for the biological functions of human cells. Increased concentration of cholesterol in humans has been associated with many diseases, including heart disease, obesity, and cancer [39–41]. An increasing number of reports have demonstrated the relationship between hypercholesterolemia and elevated ESCC risk according to clinical data [42, 43]. In addition, dysregulated cellular cholesterol levels caused by increased biosynthesis and uptake could fuel the malignant phenotypes of cancer cells, such as growth, anti-apoptosis, chemotherapy resistance, and immune escape [44–46]. Importantly, statin, a cholesterol-lowering agent, inhibited the growth of various cancer cells, including ESCC cells. Our mechanistic studies revealed that *TTPAL* silencing significantly attenuates cholesterol biosynthesis through coordinated downregulation of rate-limiting enzymes in this pathway. Pan-cancer analysis confirmed strong positive correlations between *TTPAL* expression and cholesterol biosynthetic enzyme levels across tumor types. Crucially, exogenous cholesterol supplementation restored proliferation capacity in *TTPAL*-depleted cells, establishing a causal relationship between TTPAL’s oncogenic function and cholesterol metabolic reprogramming. These results suggest that TTPAL promotes ESCC progression, which is dependent on cholesterol biosynthesis.

Cholesterol biosynthesis is an energetically expensive process; therefore, it is tightly regulated by upstream biological signaling. Three crucial players in cholesterol biosynthesis include two rate-limiting enzymes, squalene monooxygenase and 3-hydroxy-3-methylglutaryl coenzyme A reductase (HMGCR), and the master transcriptional regulator of cholesterol biosynthesis, SREBP2 [47, 48]. SREBP2 is an inactive precursor that is located in the ER membrane. To become active, SREBP2 gets cleaved into the N-terminal fragment known as nuclear SREBP2 (nSREBP2) and sequentially enters the nucleus as an active transcription factor to upregulate target gene expression. Several oncogenic signaling pathways such as AMPK, mTOR, and p53 can alter the expression of SREBP2 in cells [49]. A recent study found that STAT3 interacts with the promoter sequence of *SREBP2* and increases the expression of SREBP2 in breast cancer cells [50]. The transcriptional activity of SREBP2 is regulated by post-translational modifications. The N-terminus of SREBP2 is acetylated by the histone acetyltransferase p300, which enhances its expression and transcriptional activity. Sirtuin 1 (SIRT1) deacetylates SREBP2 and increases its abundance in the nucleus [51]. ERK and AMPK phosphorylate SREBP2 and modify transcriptional activity of SREBP2 in cells. In addition to acetylation and phosphorylation of SREBP2, sumoylation of SREBP2 decreases its transcriptional activity. In the present study, we found that TTPAL increased the stability of *SREBP2* mRNA in an m5C-dependent manner in ESCC cells. Elevated SREBP2 levels could promote the expression of target genes associated with enzymes involved in cholesterol biosynthesis to increase cholesterol concentration in ESCC cells. TTPAL may promote the progression of cholesterol biosynthesis in ESCC tissues by potentiating the TTPAL-SREBP2 axis.

Several different types of mRNA modifications, including m6A, m1A, m5C, and m7G, regulate RNA stability, subcellular localization, and mRNA translation efficiency. Different types and levels of mRNA modification occur in writers, erasers, and readers and play crucial roles in cancer initiation and progression. Accumulating evidence has confirmed that mRNA modifications participate in the regulation of cellular metabolism, suggesting new directions for cancer therapy. A recent study showed that the m6A-modification of SREBP cleave-activating protein (*SCAP*) by the m6A mRNA methyltransferase METTL3 stabilizes its mRNA, subsequently promoting cholesterol biosynthesis. The m5C is one of the most important RNA modifications in cancer cells, and is involved in multiple biological processes [52]. In the present study, we discovered that NSUN2 catalyzed the m5C modification of *SREBP2* mRNA, and ALYREF served as the “reader” of m5C modification, binding directly to *SREBP2* mRNA to stabilize it and increase the expression level of SREBP2. Subsequently, *SREBP2* activated the downstream target genes associated with cholesterol biosynthesis. We also noticed that *NSUN2* overexpression partially rescues *SREBP2* expression in *TTPAL* knockdown cells. Multiple signaling pathways, including p53, AKT, and androgen signaling, have been implicated in SREBP2 activation. Previous studies have demonstrated that TTPAL promotes gastric cancer progression through AKT pathway activation [16]. These findings suggest that TTPAL may modulate downstream effectors such as SREBP2 via AKT pathway signaling. Our current data reveal that *NUSN2* overexpression specifically enhances m5C modification of *SREBP2* mRNA, leading to increased *SREBP2* mRNA expression through post-transcriptional regulation.

Previous studies have shown that NSUN2 promotes the initiation and progression of multiple cancers via m5C modification of target genes such as *FABP5*, *TEAD1*, *PFAS*, *LIN28B*, and *HDGF* [32, 53–56]. The expression level of NSUN2 was mediated at both the transcriptional and translational levels. In ESCC, the transcription factor E2F1 binds to the promoter of *NSUN2* and elevates the expression of NSUN2, thereby activating PI3K/AKT and MAPK signaling. Recently, K356 lactylation of the NSUN2 protein was shown to be crucial for capturing target RNAs. In the present study, we explored the molecular mechanisms by which TTPAL stabilizes NSUN2 in ESCC cells. Our results showed that expression of *NSUN2* mRNA did not change in *TTPAL*-depleted or -overexpressing ESCC cells, but the NSUN2 protein level increased, suggesting that TTPAL could elevate the NSUN2 protein level by post-translational modification. TTPAL inhibited the interaction between NSUN2 and STUB1, which are NSUN2 E3 ubiquitin ligases. In summary, our results show that TTPAL relieves the ubiquitination of NSUN2 through STUB1, thereby increasing NSUN2 protein expression levels.

This study has several limitations. First, the correlation between increased *TTPAL* DNA copy number and its expression requires further validation through additional patient specimens and analysis in other tumor tissues. Second, Although we observed that the numbers of CD3 + T cells, CD19 + B cells, and F4/80 + macrophages in 4NQO-induced esophageal cancer tissues did not differ significantly between WT and *Ttpal-*KO mice, whether simvastatin affects tumor immunity in *Ttpal-*KO mice remains to be further investigated.

**Fig. 8:**
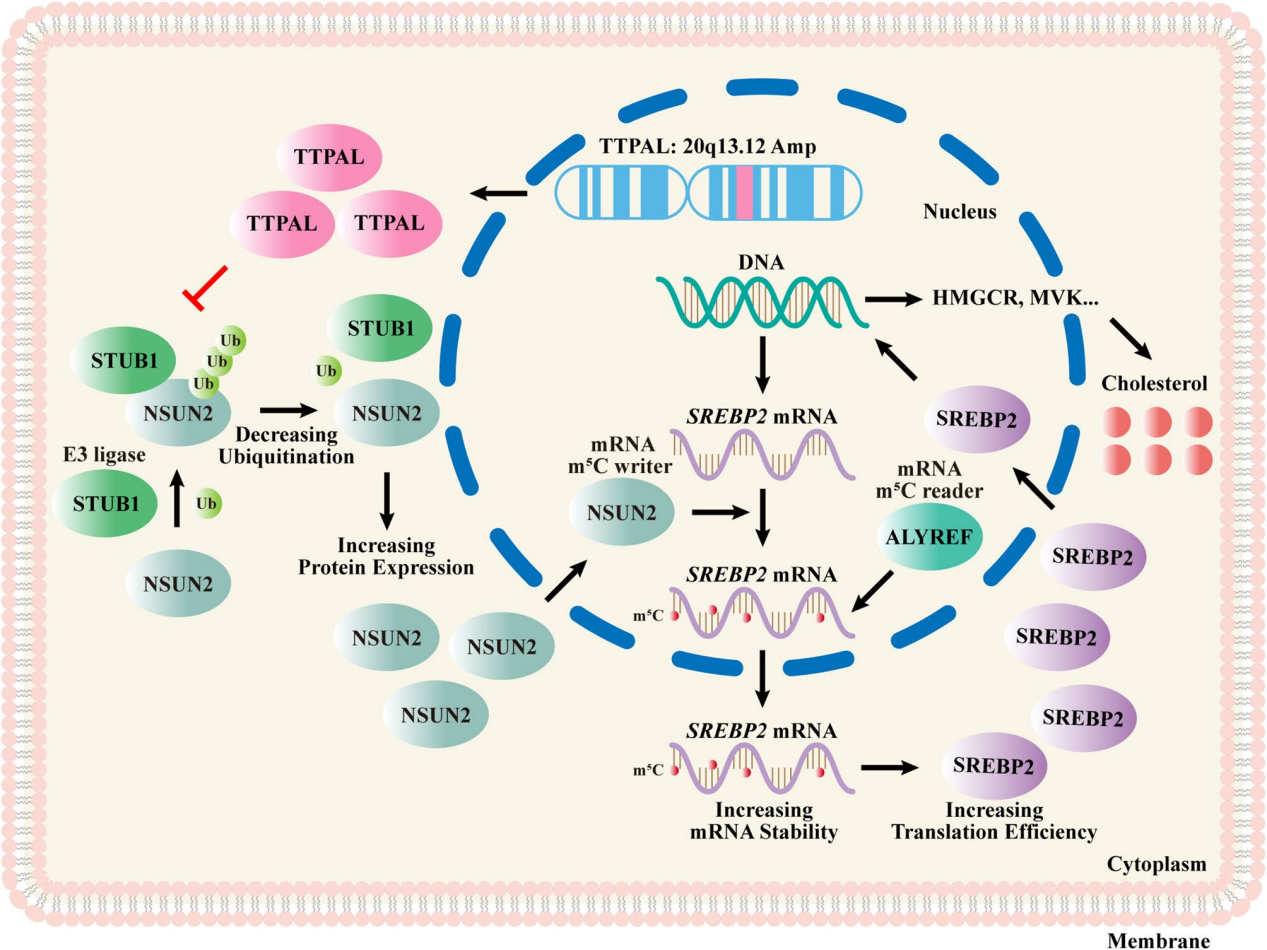
Proposed working model. In ESCC, *TTPAL* is amplified and highly expressed, resulting in relieving the ubiquitination of NSUN2 and then upregulating NSUN2 expression. NSUN2 catalyzes the m5C modification of *SREBP2* mRNA and elevates SREBP2 expression to upregulate cellular cholesterol biosynthesis and facilitate tumor progression. Inhibition of cholesterol biosynthesis with simvastatin might serve as an effective treatment method for 20q13.12-amplified and *TTPAL*-highly expressed ESCC

In conclusion, our study identifies amplification of *TTPAL* (20q13.12) as a novel oncogenic driver in ESCC pathogenesis. We establish that TTPAL promotes tumor initiation and progression through m5C-mediated stabilization of *SREBP2* mRNA, executed via its interaction with the methyltransferase NSUN2. This RNA epigenetic mechanism sustains a hypercholesterogenic state by elevating SREBP2 protein levels, which transcriptionally activates cholesterol biosynthetic enzymes to fuel ESCC proliferation and survival. Therapeutically, we demonstrate that TTPAL-high ESCCs exhibit marked sensitivity to cholesterol pathway inhibition. Pharmacological suppression using simvastatin-an FDA-approved HMGCR inhibitor-effectively reverses TTPAL-driven oncogenicity Fig. 8. Our findings position *TTPAL* amplification as both a novel biomarker for cholesterol-targeted therapies.

## Conclusion

The oncogenic role of *TTPAL* is proved in ESCC progression, providing new insights into the regulation of cholesterol biosynthesis signaling in ESCC and establishing *TTPAL* as a biomarker for simvastatin therapy resistance in ESCC.

## Electronic supplementary material

Below is the link to the electronic supplementary material.

Supplementary Material 1

## Data Availability

No datasets were generated or analysed during the current study.

## Abbreviations

4-SU: 4-thiouridine
4-NQO: 4-Nitroquinoline-1-oxide
ALYREF: Aly/REF export factor
BMDCs: Bone marrow-derived dendritic cells
BMDMs: Bone marrow-derived macrophages
CEP20: Chromosome enumeration probe 20
CHX: Cycloheximide
CNVs: Copy number variations
CQ: Chloroquine
DepMap: Cancer Dependency Map
ESCC: Esophageal squamous cell carcinoma
FFPE: Formalin-fixed paraffin-embedded
FISH: Fluorescence in situ hybridization
GM-CSF: Granulocyte-macrophage colony-stimulating factor
GWAS: Genome-wide association studies
HMGCR: 3-hydroxy-3-methylglutaryl coenzyme A reductase
IHC: Immunohistochemistry
KEGG: Kyoto encyclopedia of genes and genomes
KO: Knockout
M-CSF: Macrophage colony-stimulating factor
m5C: 5-Methylcytosine
MOI: Multiplicity of infection
NSUN1-7: NOP2/Sun RNA methyltransferase family member 1–7
NSUN2: NOP2/Sun RNA methyltransferase 2
PDX: Patients-derived xenograft
RBC: Red blood cell
RIP: RNA immunoprecipitation
SDS-PAGE: Sulfate-polyacrylamide gel electrophoresis
SIRT1: Sirtuin 1
SREBP2: Sterol regulatory element-binding transcription factor
TTPAL: Tocopherol alpha transfer protein-like
UVP: Ultraviolet Crosslinker
WB: Western blotting
WBC: White blood cell
WT: Wild type
YBX1: Y-box binding protein 1
