# Supplementary material for: Copy number amplification of TTPAL promotes cholesterol biosynthesis and esophageal squamous cell carcinoma progression via elevating NSUN2-mediated m5C modification of SREBP2 mRNA
Source: J Exp Clin Cancer Res. 2025 Jul 26;44:220. doi: 10.1186/s13046-025-03483-8 (PMC12297799; doi:10.1186/s13046-025-03483-8)
Supplement: Supplementary file 1 — Supplementary Material 1 [file 13046_2025_3483_MOESM1_ESM.docx]

**Copy number amplification of TTPAL promotes cholesterol biosynthesis and esophageal squamous cell carcinoma progression via elevating NSUN2-mediated m5C modification of SREBP2 mRNA**

Shan Huang ^#1^, Yuanyuan Liu^#2^, Manyu Zhao^#3^, Tao Wang^#4^, Lihua Mao^1^, Ting Wang^1^, Chunyuan Guo^1^, Wentao Huang^1^, Zimei Peng^5^, Zhen Zhang^5^, Rui Jiang^6^, Xingrui Ma^7^, Nimei Shen^8^, Jun Rao^*9^, Xing Wang^*1,10^, Zhi Zheng^*1^, Lixiao Chen^*2^

^1^Jiangxi Provincial People's Hospital, The First Affiliated Hospital of Nanchang Medical College, Nanchang, 330006, China.

^2^Department of Otolaryngology: Head and Neck Surgery, Shanghai General Hospital, Shanghai Jiao Tong University School of Medicine, Shanghai, 200080, China.

^3^National Engineering Research Center for Nanomedicine, Key Laboratory of Molecular Biophysics of Ministry of Education, College of Life Science and Technology, Department of Oncology, Tongji Hospital, Huazhong University of Science and Technology, Wuhan, 430074, China.

^4^Institute of Geriatrics, Jiangxi Provincial People's Hospital, The First Affiliated Hospital of Nanchang Medical College, Nanchang, Jiangxi, 330006, PR China.

^5^Institute of Clinical Medicine, Jiangxi Provincial People’s Hospital, The First Affiliated Hospital of Nanchang Medical College, Nanchang, 330006, China.

^6^Department of Otolaryngology: Head and Neck Surgery, Nanjing University of Chinese Medicine Affiliated Kunshan Traditional Chinese Medicine Hospital, Jiangsu, 215300, China.

^7^Department of Otolaryngology: Head and Neck Surgery, The First People's Hospital of Kunshan, Jiangsu, 215300, China.

^8^Department of Otolaryngology: Head and Neck Surgery, Jiangsu General Hospital, The Second Affiliated Hospital of Nantong University, Jiangsu 226000, China.

^9^Jiangxi Cancer Hospital, The Second Affiliated Hospital of Nanchang Medical College, Jiangxi Clinical Research Center for Cancer, Nanchang, 330006, China.

^10^Centre for Medical Research and Translation, Jiangxi Provincial People’s Hospital, The First Affiliated Hospital of Nanchang Medical College, Nanchang, 330006, China.

^#^These authors contributed equally.

^*^Corresponding author: [raojun1986@126.com,](mailto:raojun1986@126.com,) [qieshuidao@126.com,](mailto:qieshuidao@126.com,) zhengxia_2007@163.com. Chenlixiao1201@163.com.

**Running Title：TTPAL drives esophageal squamous cell carcinoma progression by activating cholesterol biosynthesis.**

**
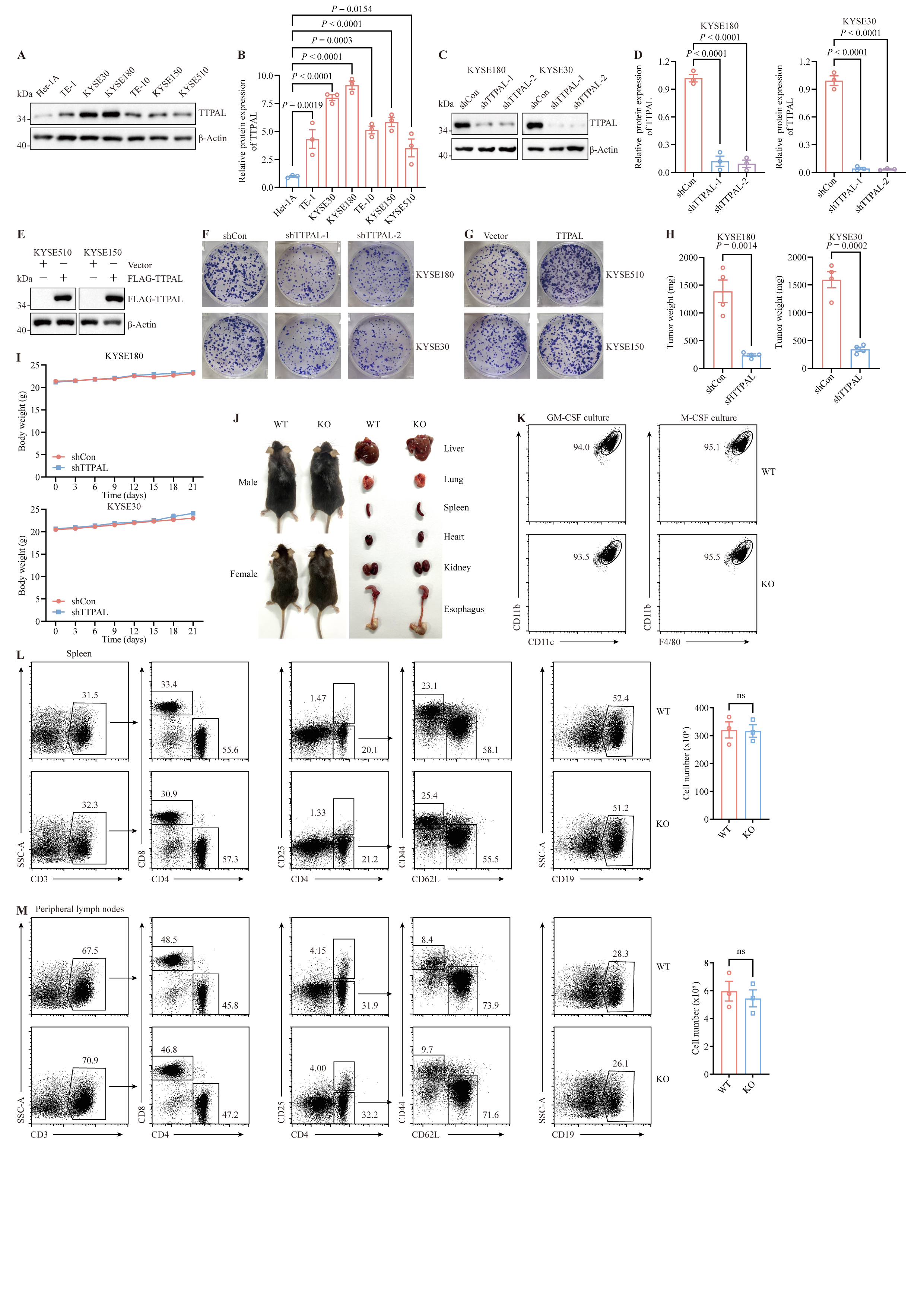
**

**Figure S1. A, B** WB analysis (**A**) and statistical quantification (**B**) of TTPAL expression in human ESCC cells (TE-1, TE-10, KYSE180, KYSE150, KYSE30, KYSE510) and non-tumorous cell line (Het1A). **C-E** WB analysis (**C, E**) and statistical quantification (**D**) of normal control (shCon) versus *TTPAL* knockdown (**C, D**), and vector versus *TTPAL*-overexpression (**E**) efficiencies in ESCC cells. **F, G** Cell colony formation was performed in *TTPAL* knockdown (**F**) or overexpression (**G**) ESCC cell lines. **H** The weight of excised subcutaneous tumors harvested by injecting shNC versus shTTPAL in KYSE180 and KYSE30 cells. **I** The body weight of mice with subcutaneous ESCC tumors. **J** The appearance and size of WT and *Ttpal*-KO mice in females and males. **K** Flow cytometry analysis of in vitro generated WT and *Ttpal*-KO mice BMDCs or BMDMs. **L, M** Flow cytometry analysis of lymphocytes and quantitative data in spleen (**L**), and peripheral lymph nodes (**M**) from WT and *Ttpal*-KO mice.


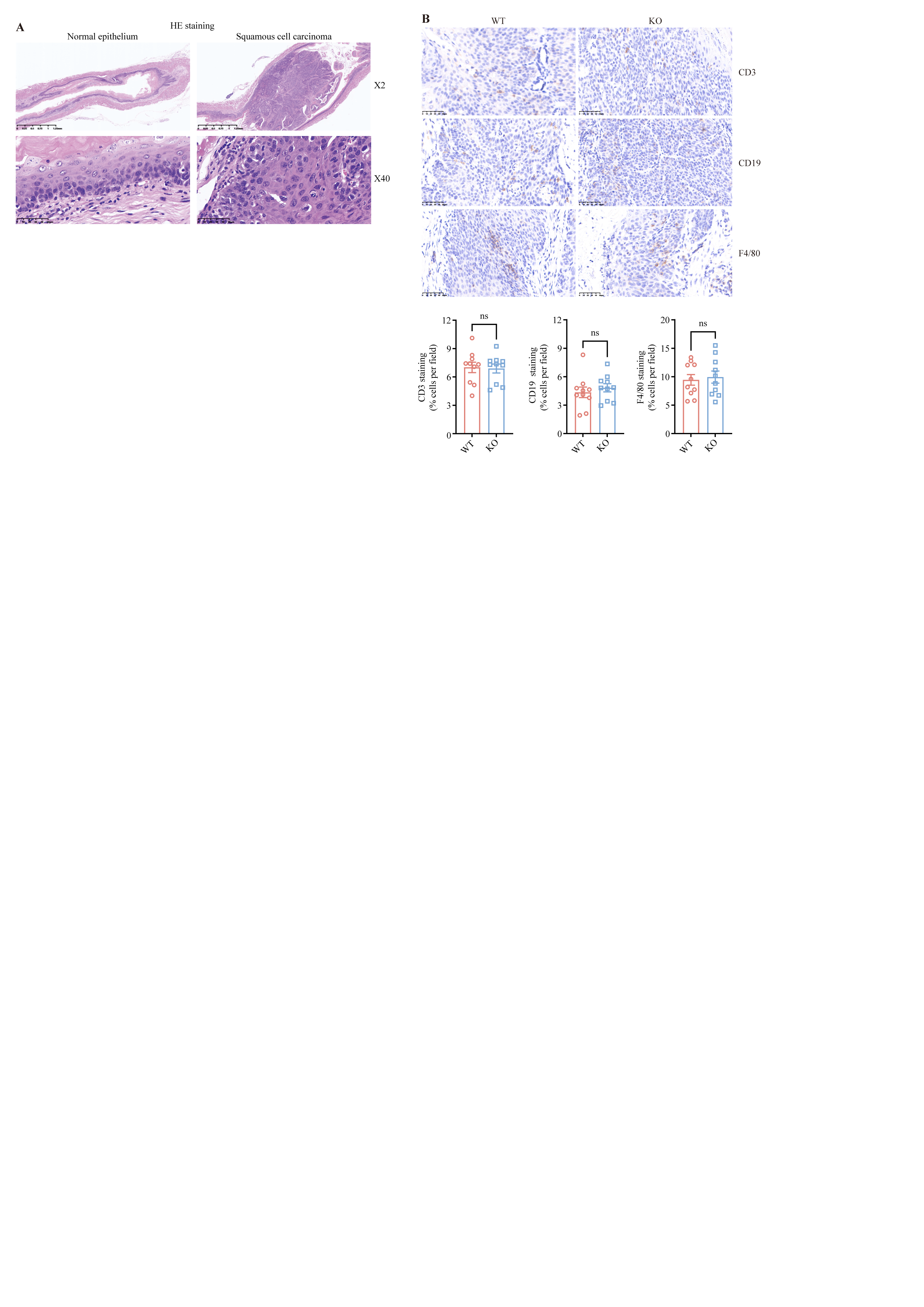
**Figure S2. A** H&E analysis of pathological features of esophagus obtained from WT mice after 4NQO withdrawal for approximately 4-8 weeks. **B** Representative immunohistochemical staining (Left) and quantitative analysis (Right) of CD3+ T cells, CD19+ B cells, and F4/80+ macrophages in esophageal cancer tissues from WT and *Ttpal-*KO mice.


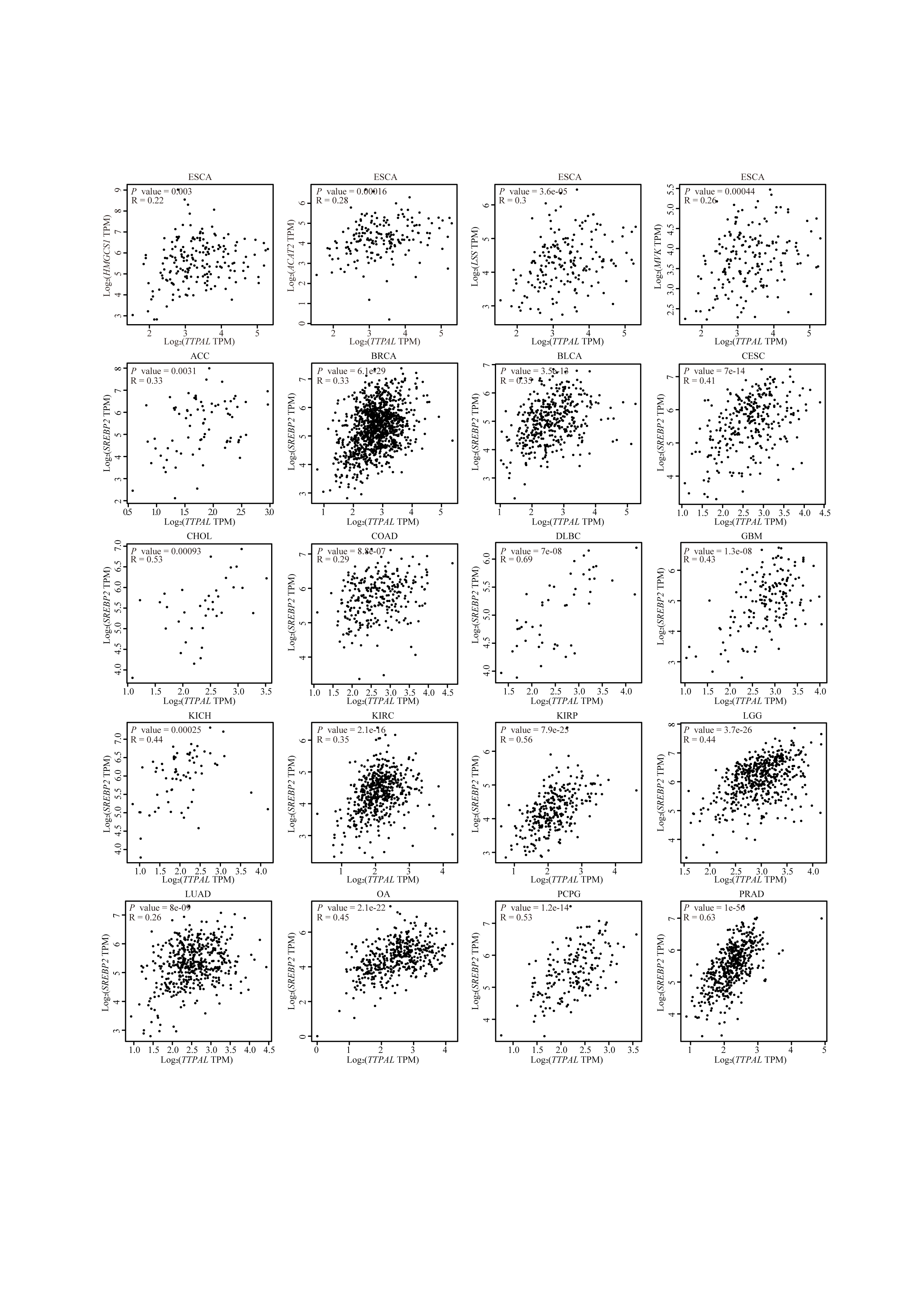


**Figure S3.** In TCGA dataset, the relationship expression of *TTPAL* with *SREBP2* and its downstream target genes in different types of cancers.


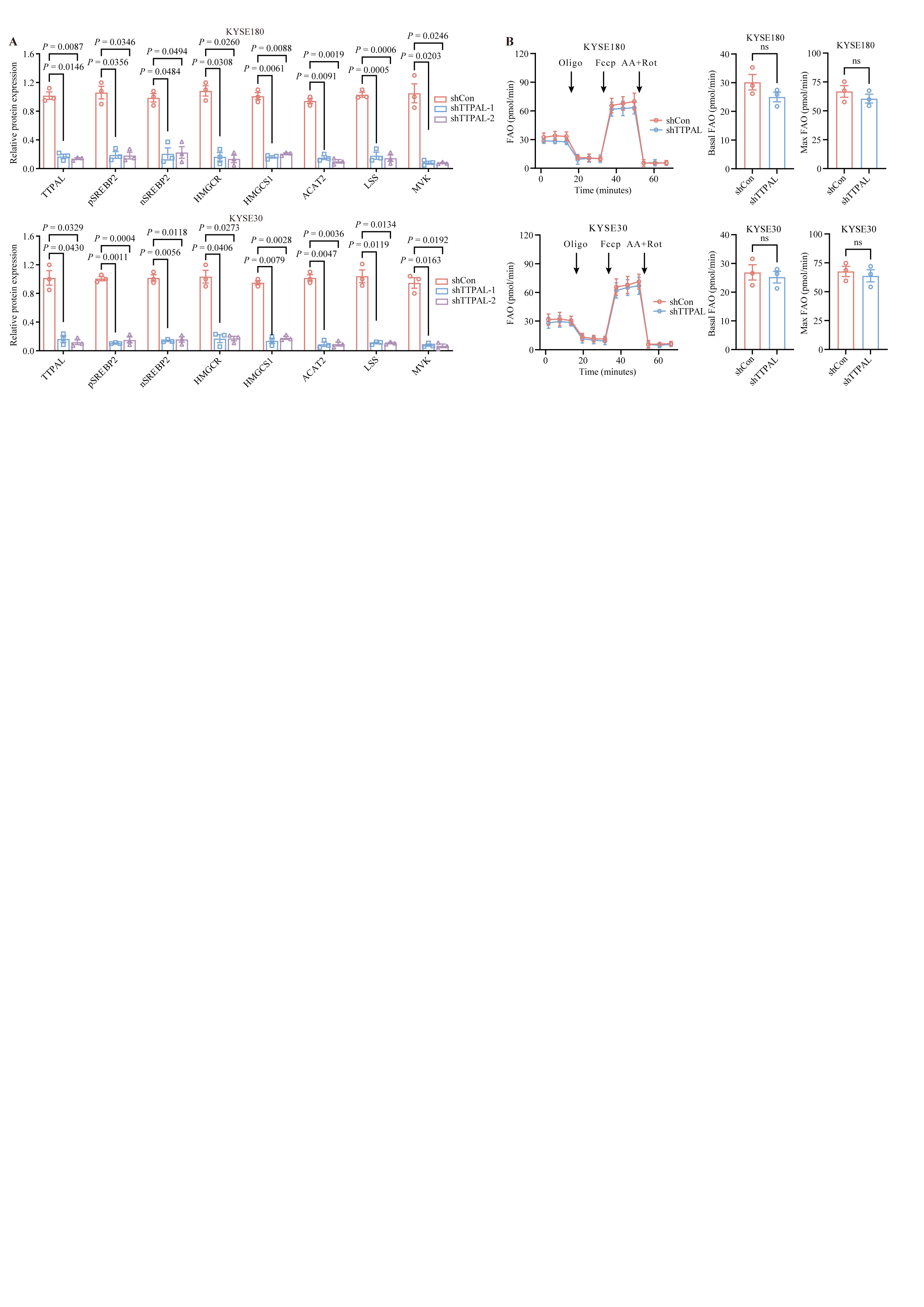


**Figure S4. TTPAL downregulates SREBP2 and its target genes without altering fatty acid oxidation (FAO). A** WB quantification of cholesterol biosynthesis pathway protein levels after *TTPAL* depletion in KYSE180 and KYSE30 cells. **B** FAO associated with *TTPAL* deficiency in ESCC cells was assessed using the Seahorse XFp Cellular Flux Analyzer.


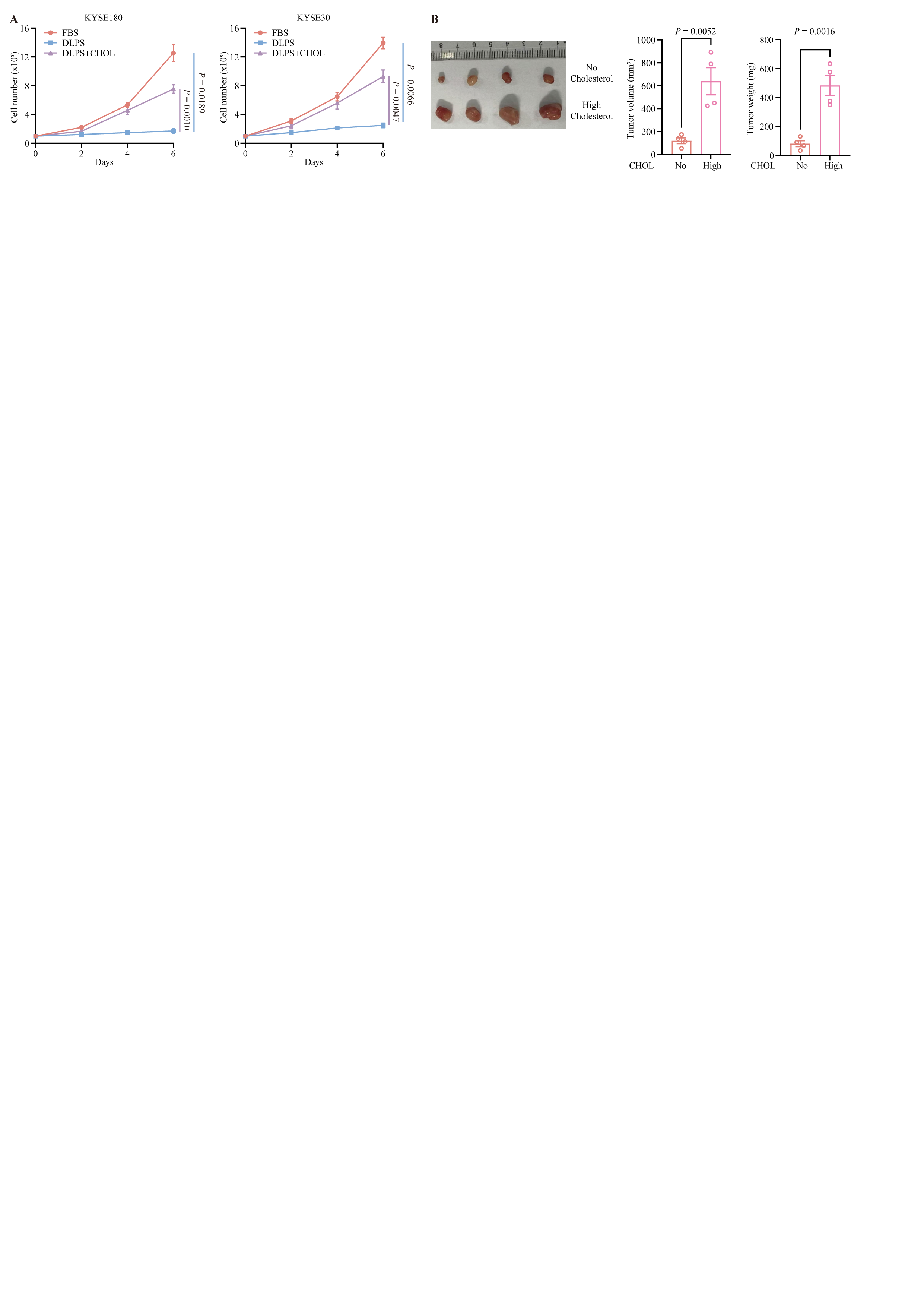


**Figure S5. Cholesterol promotes ESCC cell growth. A** The cell proliferation capacity of ESCC cells cultured in normal medium, lipoprotein-depleted FBS medium, and lipoprotein-depleted FBS medium supplemented with 5 μM cholesterol. **B** Photograph and quantification of excised subcutaneous tumors from cholesterol-free diet-fed mice versus high-cholesterol diet-fed mice.


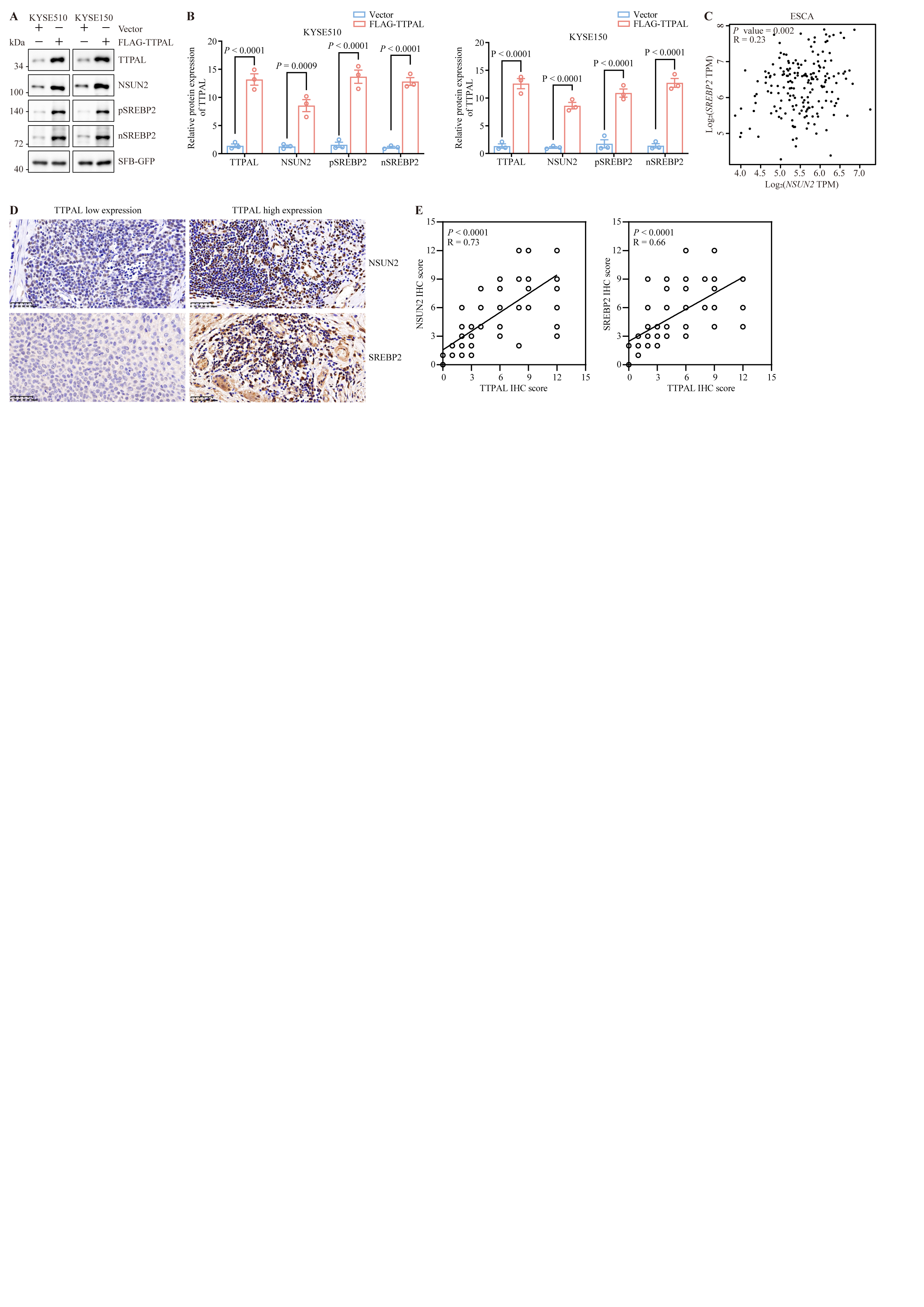


**Figure S6. TTPAL promotes NSUN2 expression in ESCC cells. A, B** WB analysis (**A**) and statistical quantification (**B**) of indicated proteins in ESCC cells with *TTPAL* overexpression. **C** In TCGA dataset, the correlated expression of *NSUN2* and *SREBP2*. **D** IHC staining of NSUN2 and SREBP2 in ESCC specimens. Representative images are shown. **E** Correlation analysis of TTPAL with SREBP2 and NSUN2 expression levels in ESCC tissues.


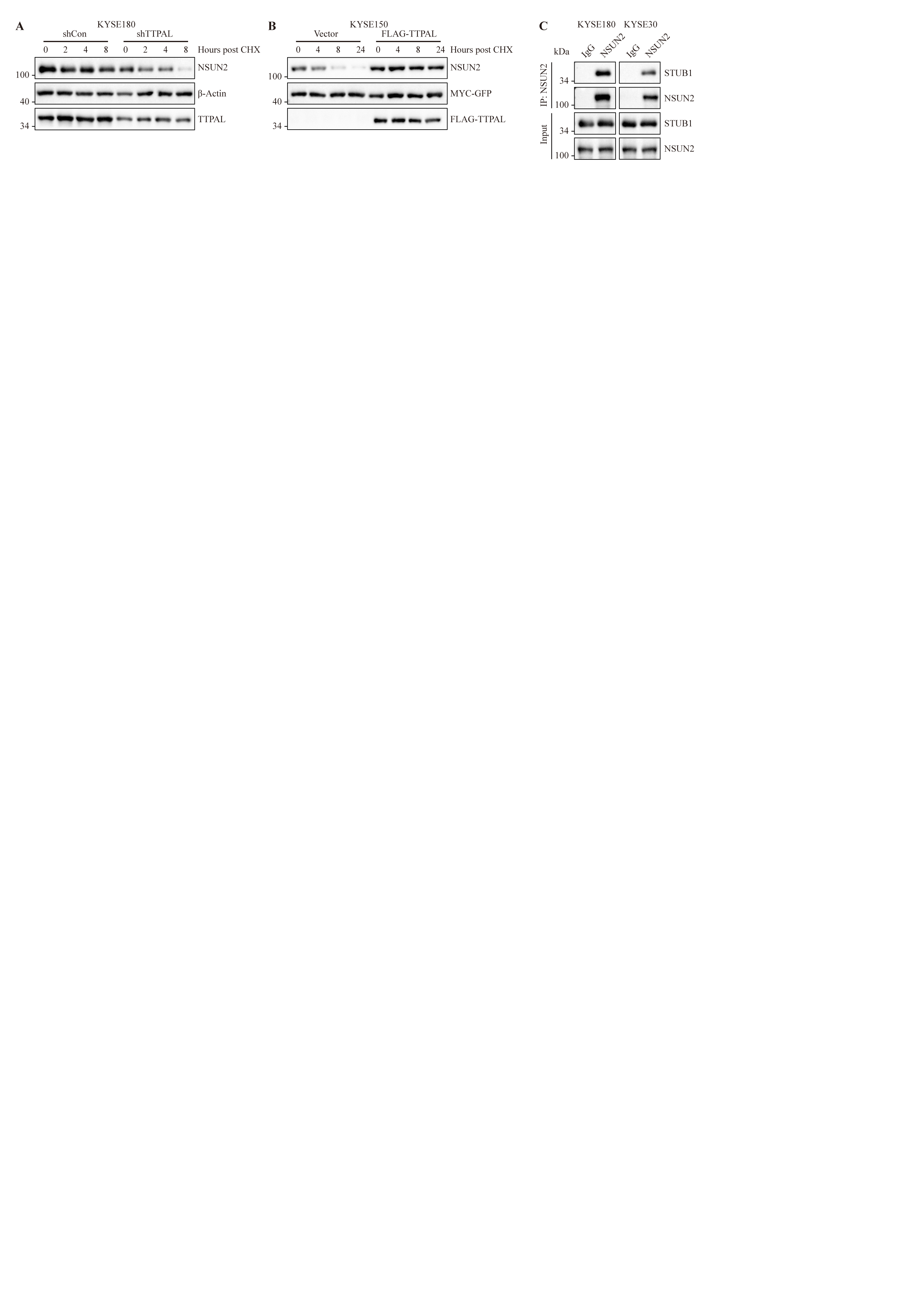


**Figure S7. TTPAL stabilizes NSUN2 by interrupting STUB1-mediated ubiquitination degradation of NSUN2 in ESCC cells. A, B** NSUN2 expression levels in ESCC cells treated with CHX for the indicated time points. **C** ESCC cells were harvested for co-IP assay by western blotting.


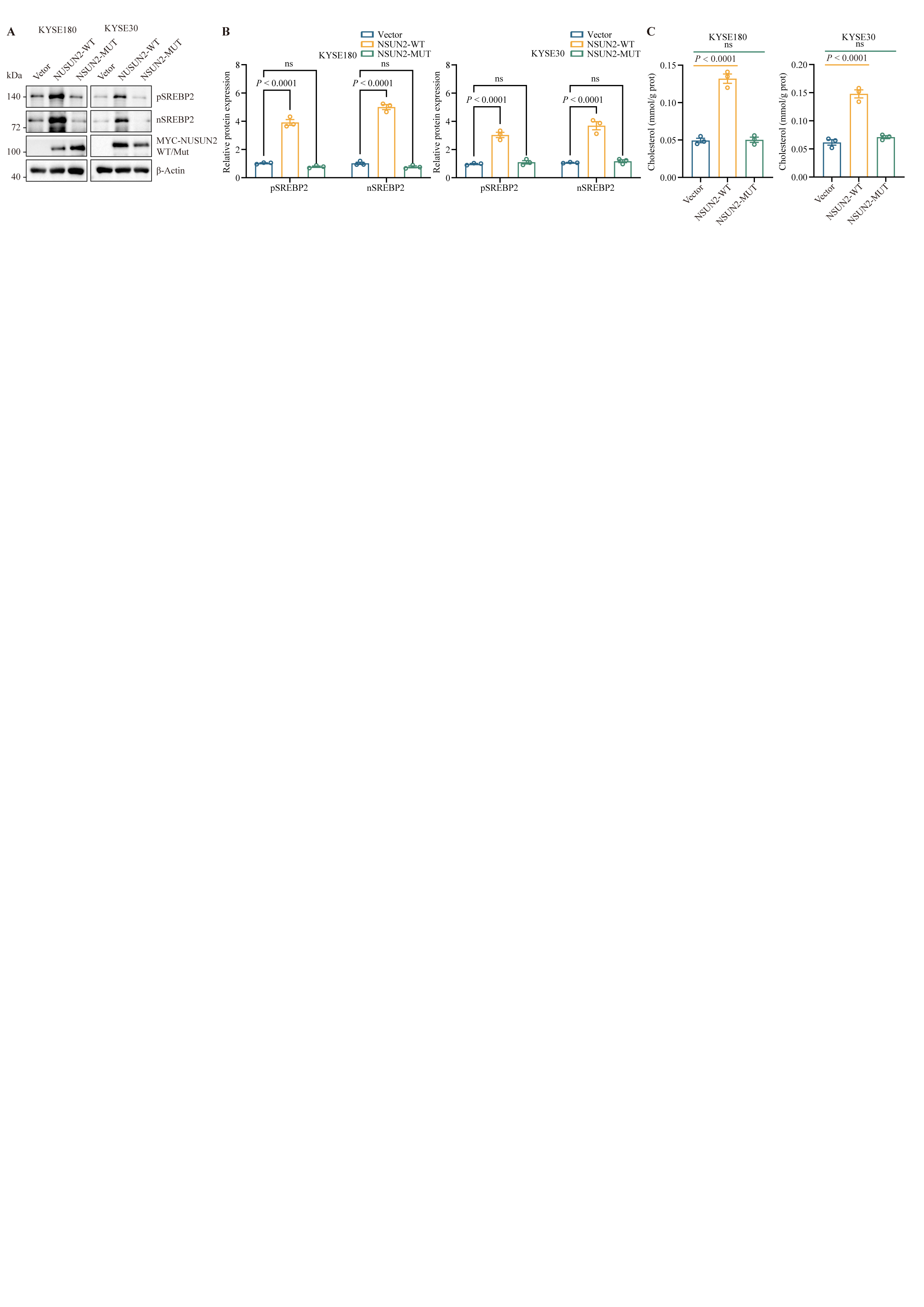


**Figure S8. NSUN2 increased SREBP2 expression and cholesterol biosynthesis. A, B** WB analysis (**A**) and statistical quantification (**B**) of indicated proteins in ESCC cells with wild type or mutant *NSUN2* overexpression. **C** Levels of total cholesterol in ESCC cells with wild type or mutant *NSUN2* overexpression.


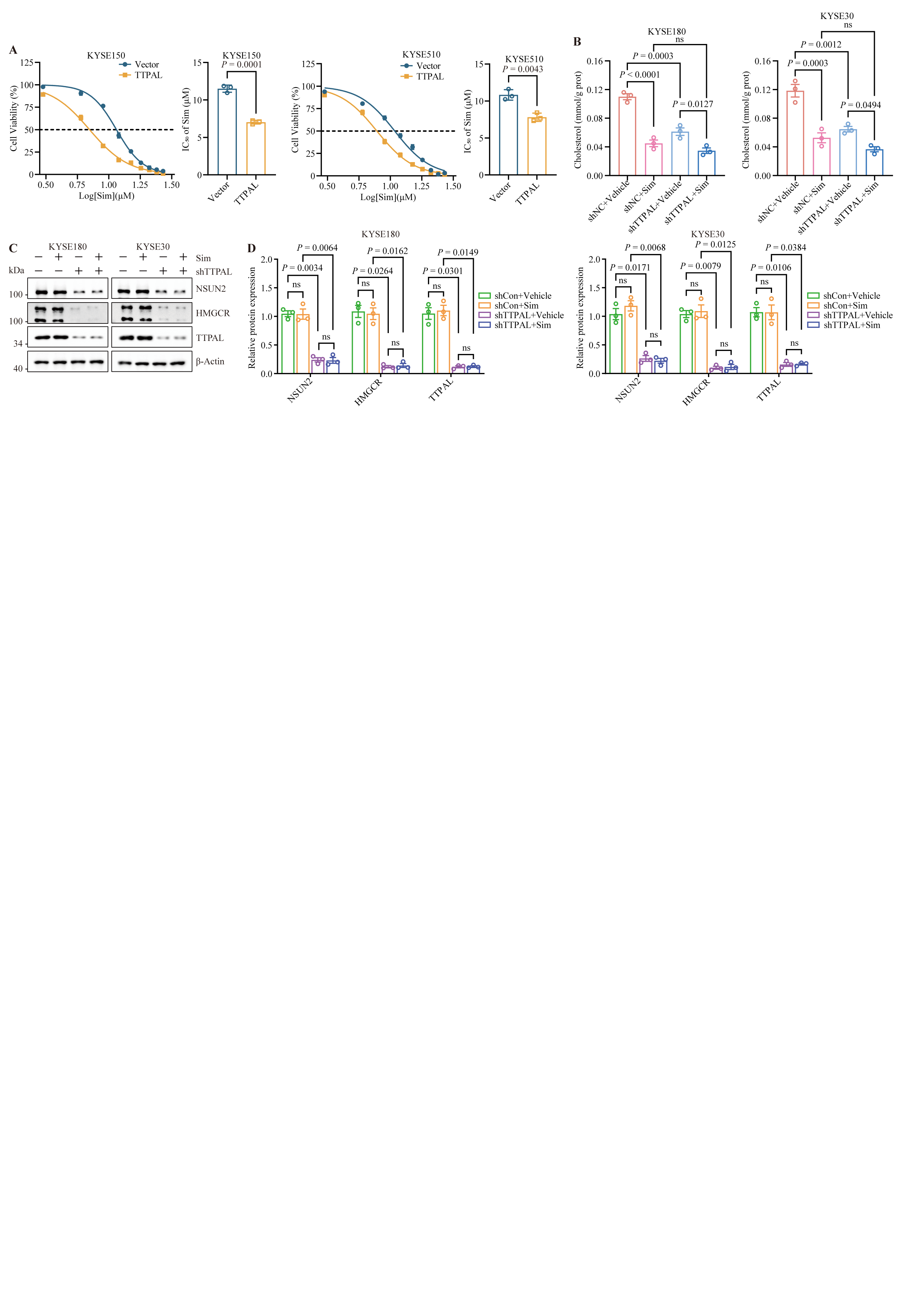


**Figure S9. TTPAL enhances simvastatin sensitivity in ESCC cells.** **A** Dose-response and IC50 values of simvastatin in KYSE510 and KYSE150 with vector or *TTPAL* overexpression. **B** Assessment of simvastatin's impact on intracellular cholesterol levels in control and *TTPAL*-knockdown KYSE180 and KYSE30 cell lines. **C, D** WB analysis (**C**) and statistical quantification (**D**) of HMGCR and NSUN2 expression levels in ESCC cells.


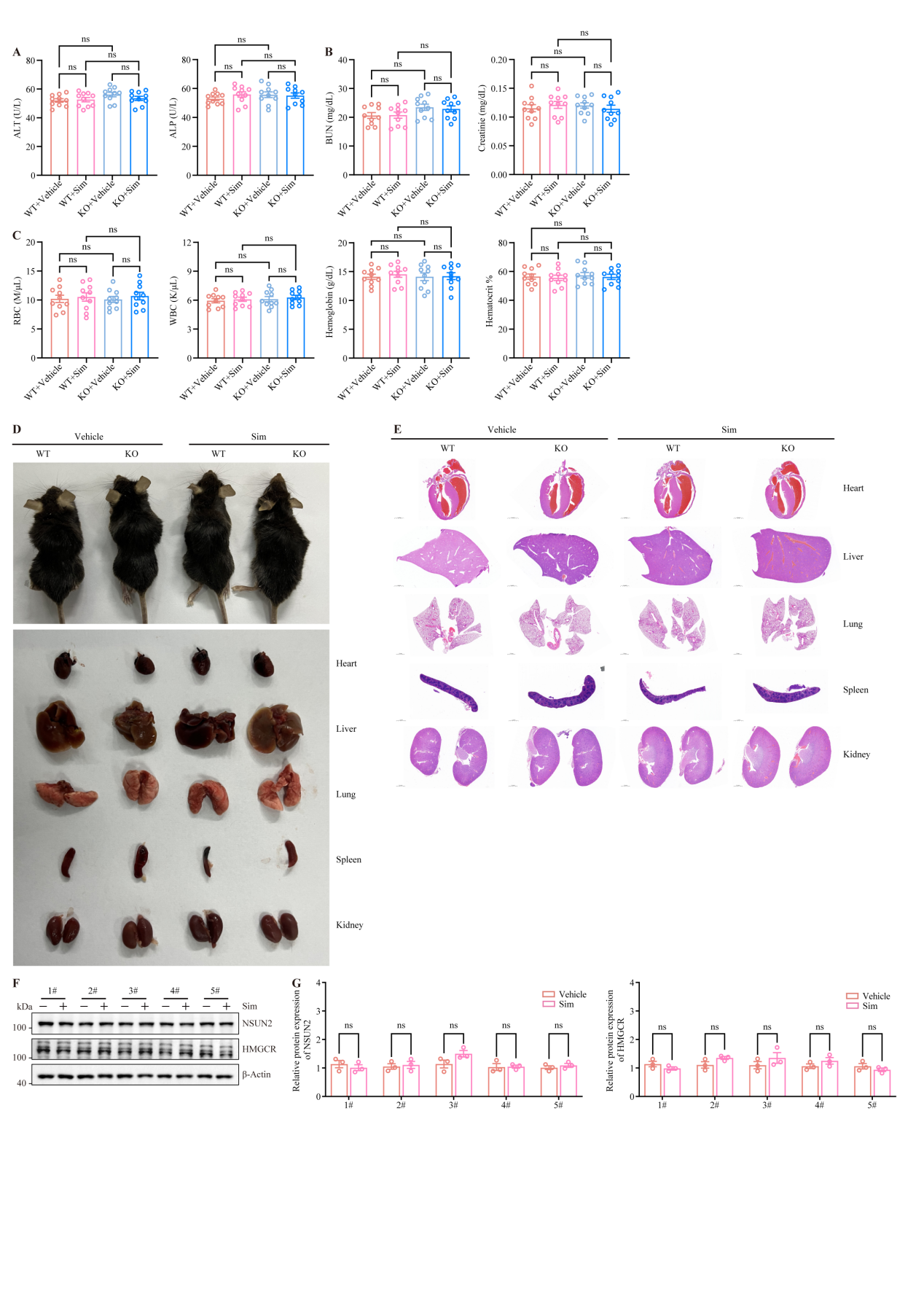


**Figure S10. Simvastatin inhibits cholesterol biosynthesis pathway in ESCC tumors without overt toxicity.** **A-C** Blood parameters involved in liver and kidney function (**A, B**) and complete cell count hematology (**C**) were assessed in 4NQO-induced WT and *Ttpal*-KO mice receiving either simvastatin treatment or vehicle control. **D,** **E** Representative images (**D**) and H&E staining (**E**) of the indicated organs in 4NQO-induced WT and *Ttpal*-KO mice receiving either simvastatin or vehicle control. **F, G** WB analysis (**F**) and statistical quantification (**G**) of HMGCR and NSUN2 expression levels in PDX tumor tissues.

**Table S1: shRNA and main primer sequences**

| Oligonucleotides | | |
| --- | --- | --- |
| shTTPAL-1: CGAGCCATATACTTGACCTTA | Sangon Biotech | N/A |
| shTTPAL-2: GCCAGTGAGAACTACTTGTAT | Sangon Biotech | N/A |
| siRBM14: CCCAAGCATCAATGGGCCTTT | Sangon Biotech | N/A |
| siRBM15: CGCGGAATACAAGACTCTGAA | Sangon Biotech | N/A |
| siIGF2BP1: ACGCTTAGAGATTGAACATTC | Sangon Biotech | N/A |
| siNSUN2: TGCAGTGTCCCATCGTCTTAT | Sangon Biotech | N/A |
| siNAT10: GCAATTGTACACAGTGACTAT | Sangon Biotech | N/A |
| siLYAP: GACAGCCAATGCAACCCTG | Sangon Biotech | N/A |
| siALYREF: CGTGGAGACAGGTGGGAAACT | Sangon Biotech | N/A |
| siYBX1: GAGAACCCTAAACCACAAGAT | Sangon Biotech | N/A |
| β-Actin-F: CATGTACGTTGCTATCCAGGC | Sangon Biotech | N/A |
| β-Actin-R: CTCCTTAATGTCACGCACGAT | Sangon Biotech | N/A |
| TTPAL-F: GGCCCTTCGTGACATGGTG | Sangon Biotech | N/A |
| TTPAL-R: CGGTCGTAATCAAACTTGCGG | Sangon Biotech | N/A |
| SREBP2-F: CCTGGGAGACATCGACGAGAT | Sangon Biotech | N/A |
| SREBP2-R: TGAATGACCGTTGCACTGAAG | Sangon Biotech | N/A |
| HMGCR-F: TTCTTGCCAACTACTTCGTGTT | Sangon Biotech | N/A |
| HMGCR-R: GCTGCCAAATTGGACGACC | Sangon Biotech | N/A |
| HMGCS1-F: CTCTTGGGATGGACGGTATGC | Sangon Biotech | N/A |
| HMGCS1-R: GCTCCAACTCCACCTGTAGG | Sangon Biotech | N/A |
| ACAT2-F: CCCAGCCAATGCTTCAGGAAT | Sangon Biotech | N/A |
| ACAT2-R: AAGCCCACGTTTATCAGCTTC | Sangon Biotech | N/A |
| LSS-F: ACATTGAGGATAAGTCCACCGT | Sangon Biotech | N/A |
| LSS-R: TCGTACCAGGTCAGGATCGTC | Sangon Biotech | N/A |
| MVK-F: GGGGATTGCGTCAACAGGT | Sangon Biotech | N/A |
| MVK-R: GGGTTCCCGTGAATCATTCTC | Sangon Biotech | N/A |

**Table S2: List of primary antibodies and their sources**

| Antibodies | SOURCE | IDENTIFIER |
| --- | --- | --- |
| TTPAL | Novus | NBP1-92544 |
| NSUN2 | Proteintech | 20854-1-AP |
| ALYREF | Proteintech | 16690-1-AP |
| Ub | Santa cruz | sc-166553 |
| STUB1 | Proteintech | 55430-1-AP |
| SREBP2 | Proteintech | 28212-1-AP |
| HMGCR | Abclonal | A1633 |
| HMGCS1 | Proteintech | 17643-1-AP |
| ACAT2 | Proteintech | 68005-1-Ig |
| LSS | Proteintech | 13715-1-AP |
| MVK | Proteintech | 12228-1-AP |
| FLAG | Absin | abs160020 |
| MYC | Absin | abs149605 |
| β-Actin | Proteintech | 20536-1-AP |
| CD3 | Proteintech | 17617-1-AP |
| CD19 | Proteintech | 31263-1-AP |
| F4/80 | Proteintech | 29414-1-AP |
| Ki-67 | Proteintech | 28074-1-AP |
